# Altitudinal patterns of plant diversity on the Jade Dragon Snow Mountain, southwestern China

**DOI:** 10.1186/s40064-016-3052-1

**Published:** 2016-09-15

**Authors:** Xiang Xu, Huayong Zhang, Wang Tian, Xiaoqiang Zeng, Hai Huang

**Affiliations:** North China Electric Power University, Research Center for Engineering Ecology and Nonlinear Science, Beijing, 102206 China

**Keywords:** Jade Dragon Snow Mountain, Altitudinal gradient, Species richness, Species-area relationship, Geometric constraint, Temperature, Precipitation

## Abstract

**Background:**

Understanding altitudinal patterns of biological diversity and their underlying mechanisms is critically important for biodiversity conservation in mountainous regions. The contribution of area to plant diversity patterns is widely acknowledged and may mask the effects of other determinant factors. In this context, it is important to examine altitudinal patterns of corrected taxon richness by eliminating the area effect. Here we adopt two methods to correct observed taxon richness: a power-law relationship between richness and area, hereafter “method 1”; and richness counted in equal-area altitudinal bands, hereafter “method 2”. We compare these two methods on the Jade Dragon Snow Mountain, which is the nearest large-scale altitudinal gradient to the Equator in the Northern Hemisphere.

**Results:**

We find that seed plant species richness, genus richness, family richness, and species richness of trees, shrubs, herbs and Groups I–III (species with elevational range size <150, between 150 and 500, and >500 m, respectively) display distinct hump-shaped patterns along the equal-elevation altitudinal gradient. The corrected taxon richness based on method 2 (TRcor_2_) also shows hump-shaped patterns for all plant groups, while the one based on method 1 (TRcor_1_) does not. As for the abiotic factors influencing the patterns, mean annual temperature, mean annual precipitation, and mid-domain effect explain a larger part of the variation in TRcor_2_ than in TRcor_1_.

**Conclusions:**

In conclusion, for biodiversity patterns on the Jade Dragon Snow Mountain, method 2 preserves the significant influences of abiotic factors to the greatest degree while eliminating the area effect. Our results thus reveal that although the classical method 1 has earned more attention and approval in previous research, method 2 can perform better under certain circumstances. We not only confirm the essential contribution of method 1 in community ecology, but also highlight the significant role of method 2 in eliminating the area effect, and call for more application of method 2 in further macroecological studies.

**Electronic supplementary material:**

The online version of this article (doi:10.1186/s40064-016-3052-1) contains supplementary material, which is available to authorized users.

## Background

Biodiversity patterns along altitudinal gradients have attracted considerable interest from ecologists over the last decade (Rahbek 2005; Guo et al. 2013; Sproull et al. 2015). Previous research has demonstrated that taxon richness commonly shows hump-shaped patterns along altitudinal gradients, i.e., taxon richness peaks at mid-elevations, for a broad spectrum of taxa (Rahbek 1995, 2005). Roughly 25 % of all published investigations have shown a monotonic decrease in taxon richness with elevation (Rahbek 1995; Kessler 2002). A few studies have observed approximately constant values from lowlands to mid-elevations, followed by a pronounced fall (Rahbek 2005; Kessler 2001). The mechanisms determining altitudinal patterns of taxon richness are still under debate (Currie 1991; Jetz and Rahbek 2002). It has been broadly recognized that area is a decisive factor shaping altitudinal taxon richness patterns (Rahbek 1997) and that the area of altitudinal bands alone could account for a large percentage of the variation in taxon richness (Bachman et al. 2004; Fu et al. 2004; Kattan and Franco 2004; McCain 2007).

Although area is admittedly important in determining altitudinal patterns of plant diversity (Rosenzweig 1995), it is also widely accepted that area is not the sole factor influencing the variation of taxon richness along the altitudinal gradient (Bachman et al. 2004; Wang et al. 2007a, b). The crucial mechanisms behind altitudinal patterns of taxon richness include the combined effects of area, climate, and the mid-domain effect (MDE; McCain 2009). Mean annual temperature (MAT) and mean annual precipitation (MAP) could also be responsible for a large part of the variance in plant taxon richness (O’Brien et al. 1998; Baudena et al. 2015). The MDE has been proposed as one important determinant for hump-shaped patterns of taxon richness along the altitudinal gradient (Colwell et al. 2004; Kluge et al. 2006). According to the MDE, species’ ranges are randomly placed within a geographical domain under the constraint that no species can exceed the hard boundaries of this domain (Colwell and Lees 2000; Colwell et al. 2004). We can simulate this stochastic procedure multiple times, and the mean number of species generated by these multiple simulations is considered to be a prediction of MDE. Some findings have shown that the combined influences of area and MDE account for virtually all variance in taxon richness along altitudinal gradients (Sanders 2002; Bachman et al. 2004). Thus, the key to understanding the underlying mechanism is disentangling the relative importance of area and MAT, MAP and MDE (Rahbek 2005; Sanders et al. 2007). However, the practical difficulty is that area, MAT, MAP, and the prediction from MDE usually covary along the altitudinal gradient (Wang et al. 2007a, b). Such collinearity might mask the possible importance of MAT, MAP, and MDE. It is therefore necessary to accurately quantify the effect of area on altitudinal patterns of plant diversity and to evaluate the effects of MAT, MAP, and MDE on the patterns after eliminating the area effect.

Species–area relationships are often applied, and are generally considered to account for area effects on altitudinal patterns of taxon richness (Rosenzweig 1995). Meza-Joya and Torres (2016) found that a power-law curve was the best-fit species–area model on the Tropical Andes and its domains. A large-scale study of vascular plant richness in North America (Qian 1998) used an exponential relationship between richness and area to eliminate the area effect on taxon richness. The species–area relationship is clearly a powerful tool for adjusting taxon richness to account for the area effect, and we refer to it as method 1 in our analysis.

Another novel yet concise method was first proposed by Bachman et al. (2004), accounting for the area effect through the use of equal-area altitudinal bands. We refer to this as method 2. Bachman et al. (2004) clarified that when assessed in equal-elevation bands, palm species richness appeared to drop monotonically with elevation, while if evaluated in equal-area bands, species richness showed a noticeable hump-shaped pattern. The reason for this was the huge percentage of lowlands in New Guinea (Bachman et al. 2004). Although this equal-area band methodology is effective in directly eliminating the area effect, it has seldom been applied to account for the area effect so far (Zhu et al. 2007; VanDerWal et al. 2008; Xing et al. 2011). Zhu et al. (2007) demonstrated that area along the equal-elevation gradient of Helan Mountain declined monotonically with elevation, and vascular plant species richness displayed hump-shaped patterns along both equal-elevation and equal-area gradients. VanDerWal et al. (2008) evaluated the effect of MDE on amphibian, bird, mammal and tree species richness along equal-area altitudinal bands in North America, and found that MDE could explain the observed pattern well for any taxa considered.

To our knowledge, the above two methods have not been implemented simultaneously for the same altitudinal gradient to date. In this study, we examine altitudinal patterns of plant diversity on the Jade Dragon Snow Mountain in southwestern China and use both methods to eliminate the area effect. The Jade Dragon Snow Mountain encompasses a broad altitudinal gradient with rich flora, diverse climate, and extensive field surveys of plant distribution over recent years (Wang et al. 2007a, b). It thus provides a valuable opportunity to explore seed plant diversity patterns along an altitudinal gradient, and the impacts of MAT, MAP, and MDE on the patterns with and without the area effect.

## Methods

### Study area and data collection

**Fig. 1:**
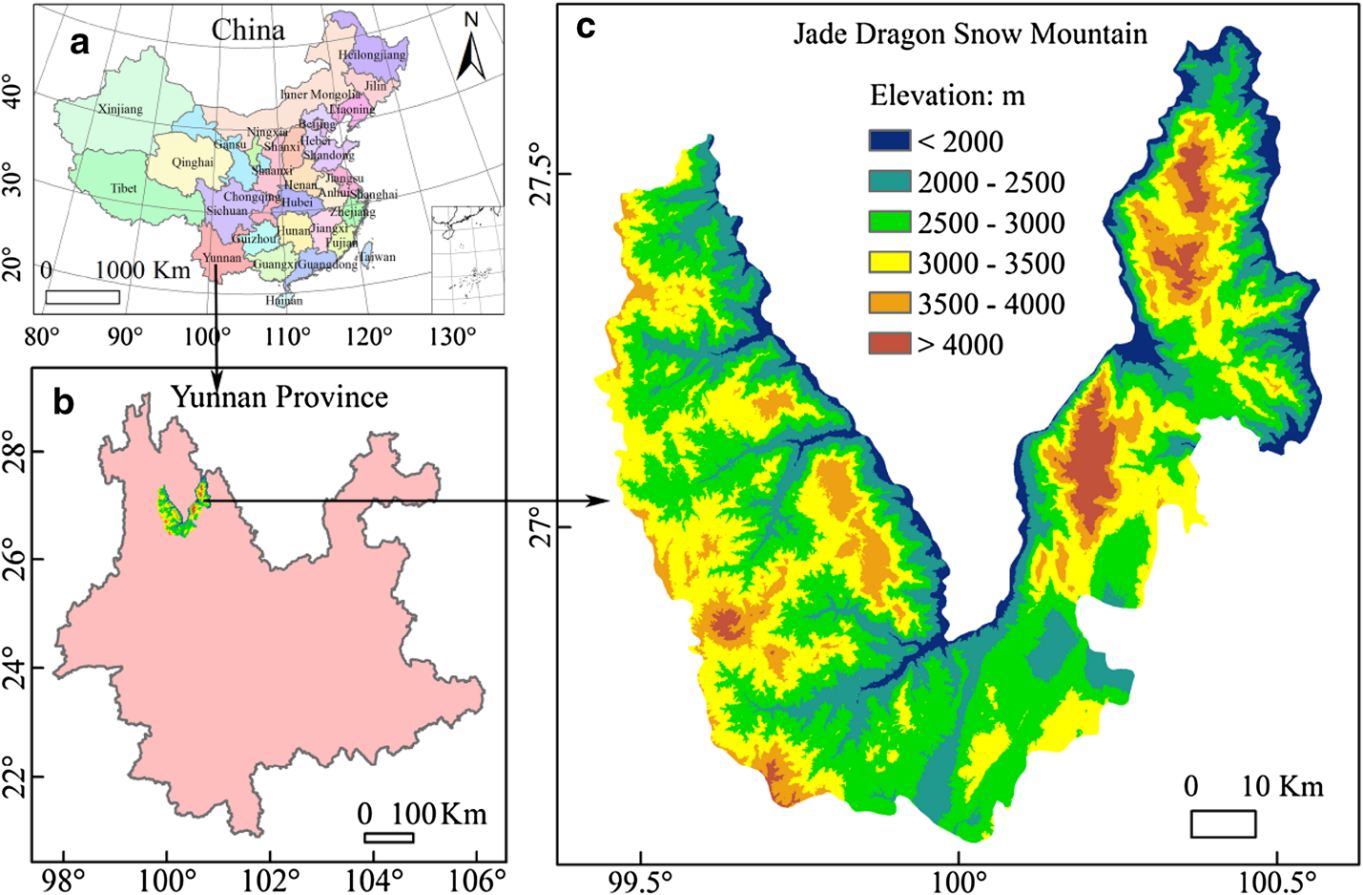
**a** Location of Yunnan province in China, **b** location of the Jade Dragon Snow Mountain in Yunnan province, and **c** topography of the Jade Dragon Snow Mountain

The Jade Dragon Snow Mountain and its adjacent region (26°35′N–27°45′N, 99°22′E–100°32′E) are located in the northwest of Yunnan Province, China, bordering the Tibetan Plateau (Fig. 1). This region covers a total area of 6127 km^2^ and has a large altitudinal gradient from 1350 to 5050 m. The climate in this region is under the control of the southwest monsoon from the Indian Ocean, with richer precipitation in the eastern part than in the western part (Wang et al. 2007a, b). Seventy percent of the MAP occurs between May and September, with winter precipitation only contributing 30 % of the annual total. The Jade Dragon Snow Mountain, with its adjacent region, is a global biodiversity hotspot (Myers et al. 2000), where the flora is rich, including a total of 2028 native seed plant species from 625 genera and 136 families. More than half of the species are herbaceous (66.4 %; 82 families; 426 genera; and 1346 species), and 33.6 % are woody species (290 tree species from 120 genera and 56 families, and 392 shrub species from 133 genera and 62 families; Wang et al. 2007a, b). The vegetation types on the Jade Dragon Snow Mountain from low to high elevations are tropical forest, subtropical forest, alpine meadow and alpine tundra, which corresponds to the general altitudinal pattern of plant diversity in northwestern Yunnan Province.

We generated a species database from the published book *Checklist of Seed Plants of Lijiang Alpine Botanic Garden* (Wang et al. 2007a, b), which is based on substantial surveys on the Jade Dragon Snow Mountain and in its adjacent region. This species database includes minimum and maximum elevations of occurrence for each species, and life-form (tree, shrub, and herb) information on each species. We extracted topographical data from ASTER GDEM V2 (Global Digital Elevation Model Version 2, DOI:10.5067/ASTER/ASTGTM.002) with 30 m × 30 m resolution. We obtained MAT and MAP data from the WorldClim database (Hijmans et al. 2005), which is frequently used in ecological studies (e.g., Sommer et al. 2010).

### Equal-elevation altitudinal gradient

**Fig. 2:**
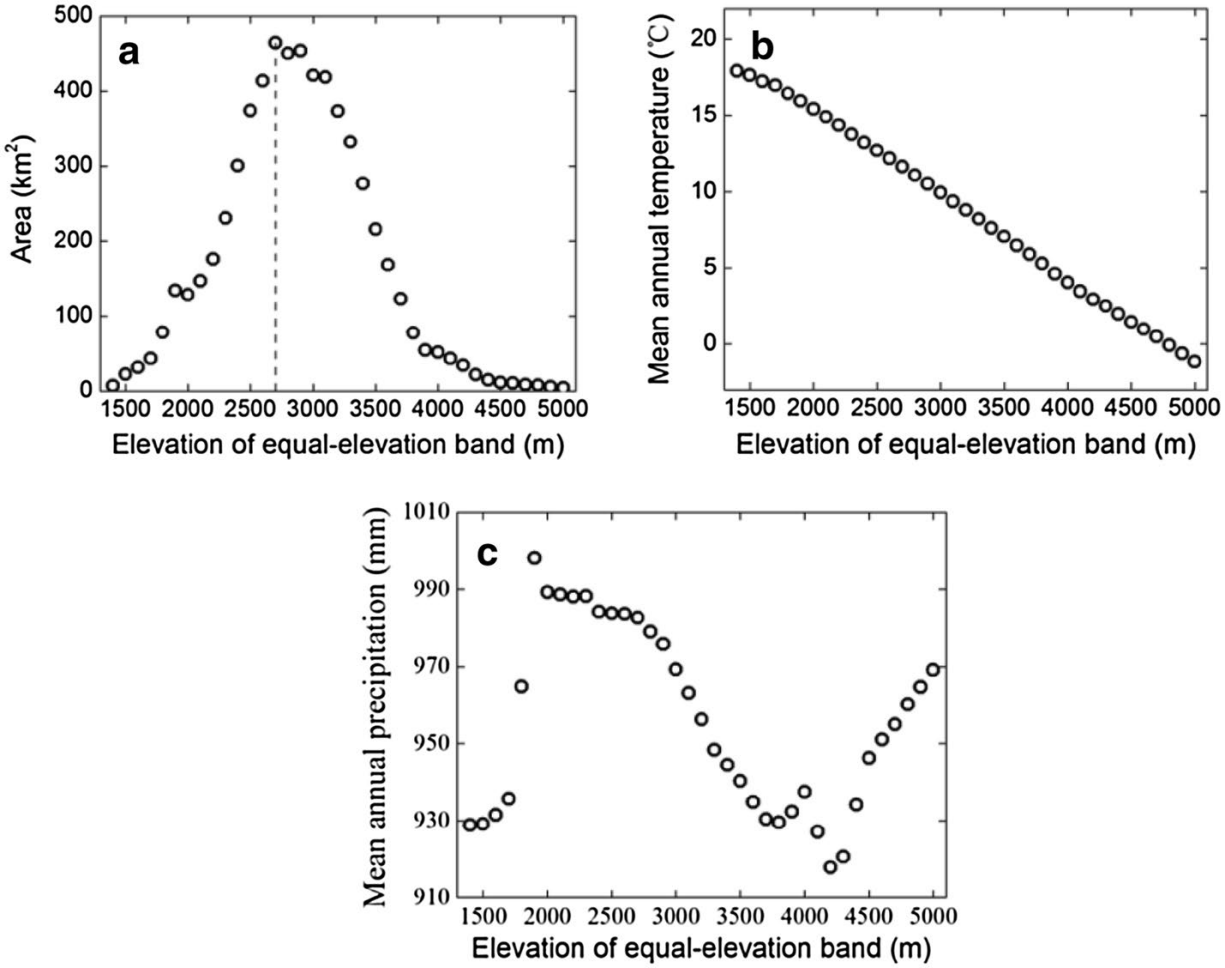
Scatter plots showing the variation in **a** area, **b** mean annual temperature, and **c** mean annual precipitation along the equal-elevation altitudinal gradient. The *dashed line* indicates the elevation at maximum area

We divided the Jade Dragon Snow Mountain into 37 100-m altitudinal bands from 1350 to 5050 m. Along this equal-elevation gradient, we calculated the area of each altitudinal band by multiplying 900 m^2^ by the number of digital elevation model (DEM) grids in each band. The area increases steeply with increasing elevation, and then decreases above the 2650–2750 m altitudinal band, showing a hump-shaped pattern (Fig. 2a). MAT declined monotonically with elevation (Fig. 2b). MAP illustrated a much more complex pattern with elevation, increasing below 1900 m, then declining steeply to 912.0 mm at 4200 m and afterward increasing to the top of the Jade Dragon Snow Mountain (Fig. 2c).

We assumed that each species had a continuous distribution range between its recorded minimum and maximum elevations, as widely used in previous studies (e.g., Rahbek 1997; Vetaas and Grytnes 2002; Sanders 2002). However, among the 2028 seed plant species, there are 717 species that have been recorded only in a single elevational band. We counted the number of seed plant species, genera, and families present at each altitudinal band as observed species, genus and family richness (Sobs, Gobs, and Fobs). To explore altitudinal patterns of plant diversity for different life-forms, we counted the number of tree, shrub, and herb species occurring at each altitudinal band as the species richness for trees, shrubs, and herbs (TSobs, SSobs, and HSobs). Additionally, in order to evaluate whether the altitudinal biodiversity patterns and their determinant predictors were dependent on species’ range size, we divided all species into three groups according to their altitudinal range sizes: <150 m (Group I, 830 species), between 150 and 500 m (Group II, 554 species), and >500 m (Group III, 644 species). These range size limits were chosen to make the number of species in each group comparable. In the same way, we obtained the species richness for Groups I–III (ISobs, IISobs, IIISobs) by counting the number of species in Groups I, II, and III present in each altitudinal band. We thus generated nine observed taxon richness variables (hereafter TRobs): Sobs, Gobs, Fobs, TSobs, SSobs, HSobs, ISobs, IISobs, and IIISobs.

### Effect of interpolation

Like many previous studies, we considered a species to be present at all elevations within its recorded elevation limits (Kluge et al. 2006). However, this approach may produce artificially elevated species richness at mid-elevations, since such interpolated data are disproportionately added to mid-elevations as opposed to edges of the gradient (Karger et al. 2011). In order to find out whether using interpolated species richness masks its real pattern along the altitudinal gradient, we evaluated the relationship between elevation and species richness of a particular plant species set (717 species that have been recorded only in a single elevational band) without interpolation (Vetaas and Grytnes 2002). We checked whether this species richness pattern generated without interpolation manifested a hump-shaped curve. If it indeed shows a hump-shaped pattern, we can conclude that the effect of interpolation may not be essential for the hump-shaped pattern of plant species on the Jade Dragon Snow Mountain.

### Species–area relationship

The species–area relationship has been universally acknowledged, but the exact structure of the relationship is still under discussion (Connor and McCoy 1979; Crawley and Harral 2001). Three common versions of the species–area relationships are: untransformed (species richness versus area), semi-log (species richness versus log area), and log–log (log species richness versus log area) transformed (Matthews et al. 2014). We conducted linear fittings to all three versions using ordinary least square (OLS) regression models, which was commonly used for studies on the species-area relationships (Matthews et al. 2014). To select the version (i.e., untransformed, semi-log or log–log) with the best performance, we calculated the modified Akaike information criterion (AIC_C_) corrected for small samples as follows:

1 $$AIC_{C} = - 2 \times \log {\text{Lik}}\left( {\text{model} } \right) + 2K\frac{n}{n - K - 1},$$

where *n* is the number of samples and *K* the number of parameters in the model. We considered the model with the lowest AIC_C_ score to be the best model, which is consistent with previous studies (Matthews et al. 2014). Then we compared the difference between the AIC_C_ of each model and the minimum AIC_C_ found, and we refer to this difference as ∆(AIC_C_). Any model with ∆(AIC_C_) < 2 is reported to be as good as the best model (Burnham and Anderson 2002). This analysis was performed using the function ‘AICc’ within the ‘AICcmodavg’ package (Mazerolle 2011) in R 2.14.2 (R Core Team 2014). To further assess the performances of linear fits, we also measured the adjusted coefficients of determination and conducted the significant F-test (Crawley 2002).

The log–log transformed species–area relationship was found to be the best-fit model (Δ(AIC_C_) > 2) for all plant groups (Additional file 1: Table S1; Figures S1–S3). We thus chose the log–log transformed version (i.e., power-law) to correct TRobs. This power-law model, S = *c*A^*z*^, where S is TRobs, *z* is a constant describing the slope of the species–area relationship in the log–log transformed space (Additional file 1: Fig. S3), A is the area of elevational bands along the equal-elevation gradient and *c* is the area-corrected taxon richness (hereafter TRcor_1_). In order to rescale TRcor_1_ to similar values as TRobs, TRcor_1_ were calculated as 100(TRobs/A^*z*^).

### Equal-area altitudinal gradient

**Fig. 3:**
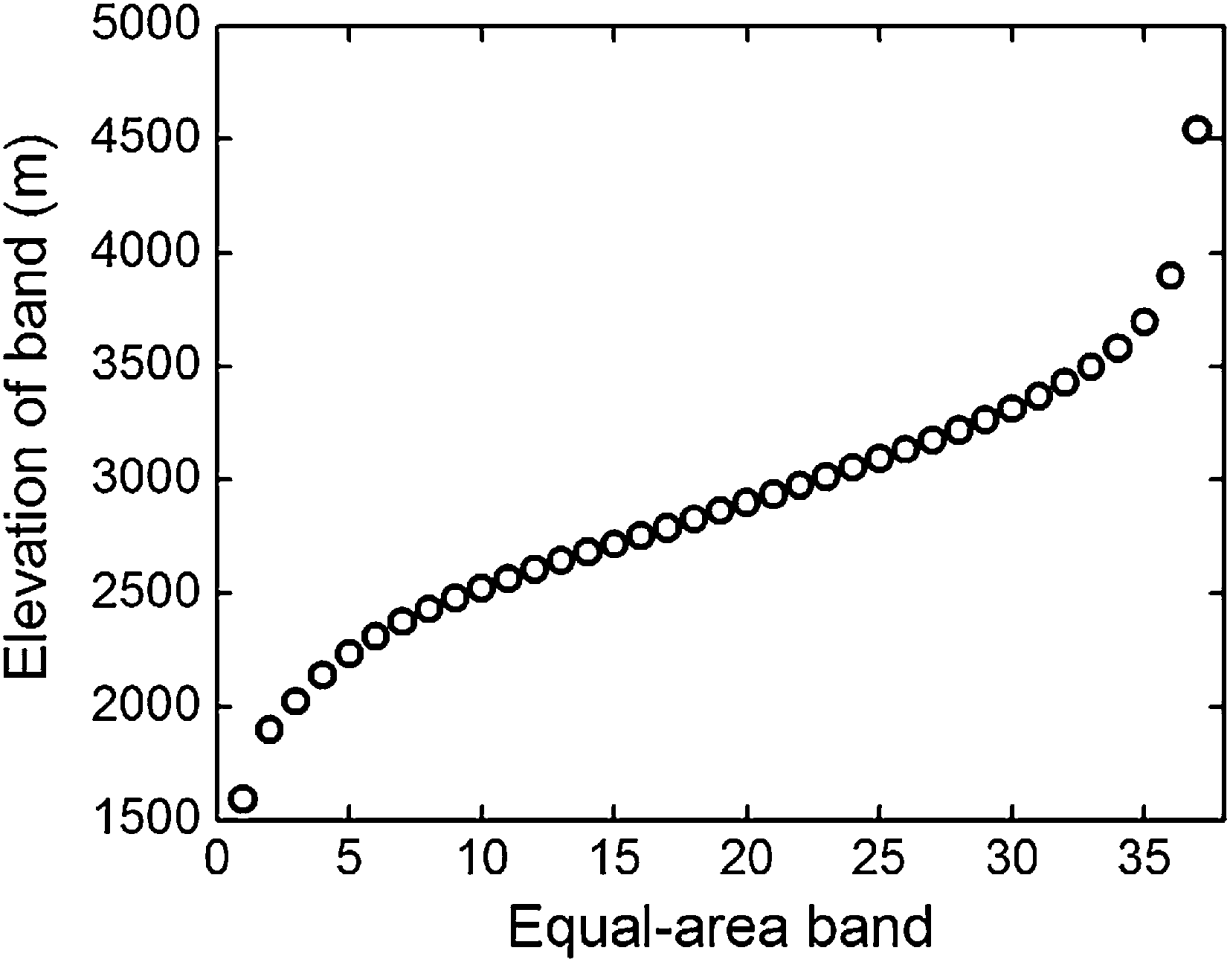
The relationship between middle elevation of each equal-area band and band sequence number

**Fig. 4:**
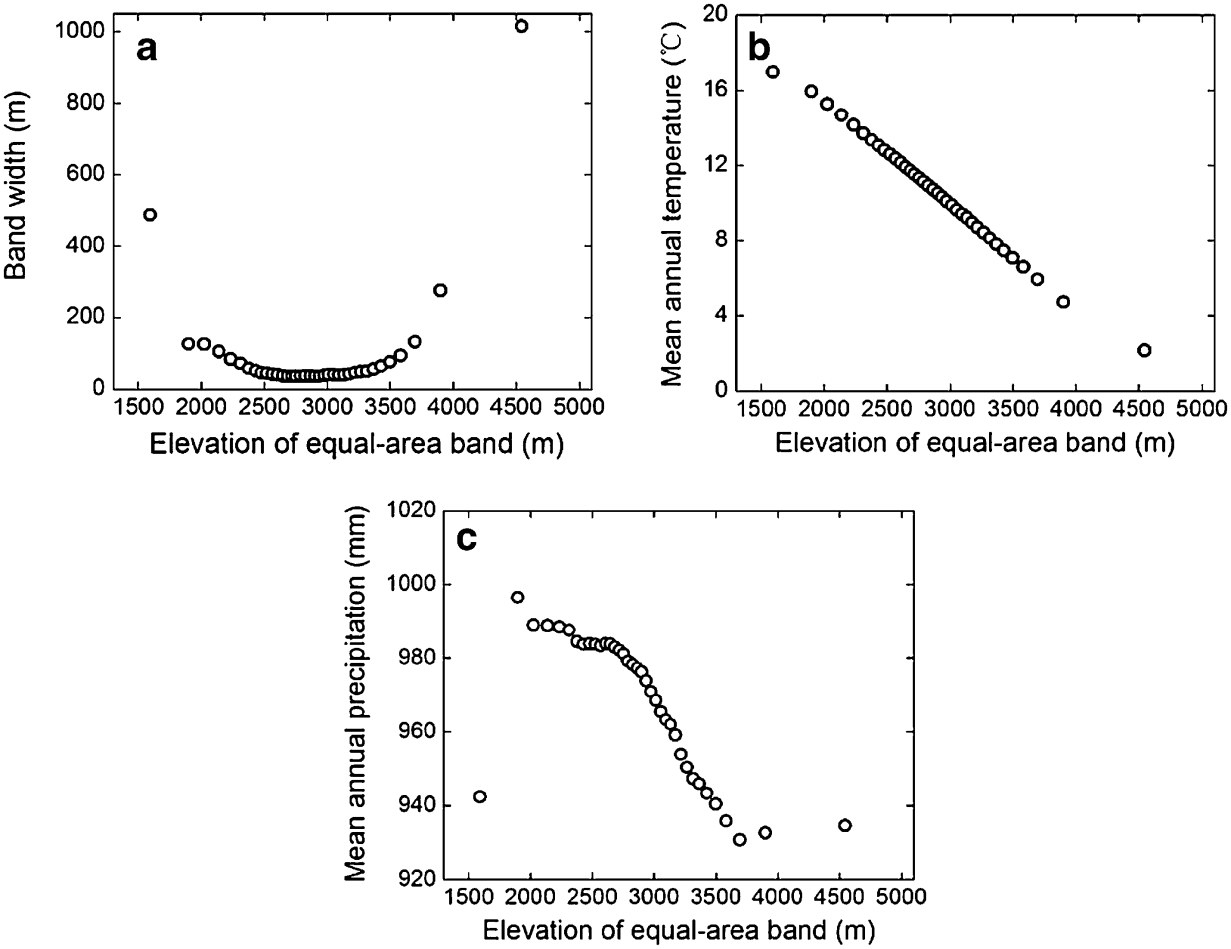
Scatter plots showing the variation in **a** altitudinal band width, **b** mean annual temperature, and **c** mean annual precipitation along the equal-area altitudinal gradient

Another approach to account for area effect was introduced by Bachman et al. (2004), and we refer to this as method 2 in our analysis. The original DEM cell values are integers and were added a random number between −0.5 and +0.5, as done in previous studies (Bachman et al. 2004). This produced DEM cells are easily classified into 37 equal-area altitudinal bands from 1350 to 5050 m using software ArcGIS version 9 (Environmental Systems Research Institute, Redlands, CA, USA). The middle elevations of the equal-area bands were not uniformly distributed along the altitudinal gradient (Fig. 3). The altitudinal band width first decreased then increased as elevation rose (Fig. 4a). The band with the smallest width (35.4 m) was the 15th, while the largest width was 1014.1 m for the 37th band. MAT still declined monotonically, and MAP showed a similar but simpler pattern along the equal-area gradient compared with the equal-elevation gradient (Figs. 2b, c, 4b, c). Along the equal-area altitudinal gradient, we counted the number of seed plant species at each band as Scor_2_. In the same way, we got Gcor_2_, Fcor_2_, TScor_2_, SScor_2_, HScor_2_, IScor_2_, IIScor_2_, and IIIScor_2_ for other plant groups. All these nine area-corrected taxon richness variables were collectively called TRcor_2_.

### Mid-domain effect

We used RangeModel (Colwell 2008) to generate the simulated taxon richness, which are the mid-domain null model predictions. This software placed empirical species ranges within the domain (i.e. the mountain range from the lowest elevation to the peak) randomly and under the constraint that no species extended beyond domain boundaries (Colwell and Lees 2000; Colwell et al. 2004). Then the number of species was counted within each elevational band. We conducted 1000 simulations, and the mean of these simulations was called a prediction from MDE. This procedure was carried out along the equal-elevation and equal-area gradient.

### Statistical analysis

We first conducted OLS regressions between taxon richness (TRobs, TRcor_1_, and TRcor_2_) and each explanatory variable (elevation, MAT, MAP, and prediction from MDE), respectively (Additional file 1: Figures S4–S6). In order to compare the performances of first- and second-order polynomial regressions, we calculated ∆(AIC_C_) according to Eq. (1). Model with the minimum AIC_C_ and ∆(AIC_C_) > 2, was selected as the best one. Two models with ∆(AIC_C_) < 2 were considered to have the same good performance, and in this case, the first-order polynomial regression was selected as the best model for simplicity (Additional file 1: Tables S2–S4).

Secondly, we examined the spatial autocorrelation in the residuals of the best OLS model using Moran’s *I* coefficient. We regard OLS model residuals in the 37 sequential elevational bands (from 1350 to 5050 m) as 37 observations along a single geographic axis. Moran’s *I* coefficients are computed from pairs of observations found at preselected distances: distance = 100 m, 200 m, 300 m, etc. (Legendre and Legendre 1998). Moran’s *I* coefficient is defined as follows:

2 $$I = \frac{n}{S}\frac{{\sum\nolimits_{i = 1}^{n} {\sum\nolimits_{j = 1}^{n} {w_{ij} (x_{i} - \bar{x})(x_{j} - \bar{x})} } }}{{\sum\nolimits_{i = 1}^{n} {(x_{i} - \bar{x})^{2} } }},$$

where *n* is the number of elevational bands, *x*_*i*_ and *x*_*j*_ represent observations in elevational bands *i* and *j*, $\bar{x}$ is the mean of all *x*, and *w*_*ij*_ is an element in the (*n* × *n*) weighting matrix *W*. It can be given as follows:

3 $$w_{ij} = \left\{ {\begin{array}{*{20}l} 1 \hfill &\quad {i,j\,{\text{is found at a given distance}}} \hfill \\ 0 \hfill &\quad {\text{otherwise}} \hfill \\ \end{array} } \right.,$$

*S* represents the sum of the weights *w*_*ij*_ (i.e., the number of connections in the matrix *W*) as follows:

4 $$S = \sum\limits_{i = 1}^{n} {\sum\limits_{j = 1}^{n} {w_{ij} } } ,$$

Moran’s *I* coefficient varies between −1.0 and 1.0 for maximum negative and positive spatial autocorrelation, respectively. Non-zero values of Moran’s *I* coefficient indicate that observations in elevational bands connected at a given distance are more similar (positive autocorrelation) or less similar (negative autocorrelation) than expected by chance. Through plotting Moran’s *I* coefficients against the preselected distances, we constructed spatial correlograms and detected considerable spatial autocorrelation in OLS model residuals (Additional file 1: Figures S7–S18). This analysis was performed using function ‘correlog’ within the package ‘ncf’ (Bjørnstad 2006).

However, spatial autocorrelation has been reported to inflate Type I errors and thus lead to the biased model comparison and poor parameter estimates, through violating assumptions of independence and identical distribution of model residuals (Dormann et al. 2007). Therefore, thirdly we ran simultaneous autoregressive (SAR) models of the error type (Kissling and Carl 2007) to correlate taxon richness (TRobs, TRcor_1_, and TRcor_2_) and each explanatory variable (elevation, MAT, MAP, and prediction from MDE), respectively. Spatial weights matrices in SAR were based on row standardization and neighborhood distance of 100 m. Pseudo-*R*^2^ values of SAR were calculated as the squared Pearson product-moment correlation coefficient between observed and predicted values (Kissling and Carl 2007). We implemented SAR models with package ‘spdep’ (Bivand 2014). We also performed first- and second-order polynomial SAR models and compared their performances using ∆(AIC_C_). The selection criterion of the best model was exactly the same as OLS analysis. The best SAR models reduced spatial autocorrelation in the model residuals to a lower level, especially at the distance of 100 m (Additional file 1: Figures S7–S18).

There is no consensus on which area-correction method is better. Each method has its advantages and drawbacks. In this study, we try to find which area-correction method preserves the influences of other factors to a larger degree after eliminating the area effect. Therefore, fourthly we compared SAR pseudo-*R*^2^ values of method 1 and 2, and consider the method with the larger pseudo-*R*^2^ values to have the better performance.

## Results

### Altitudinal patterns of observed taxon richness

**Fig. 5:**
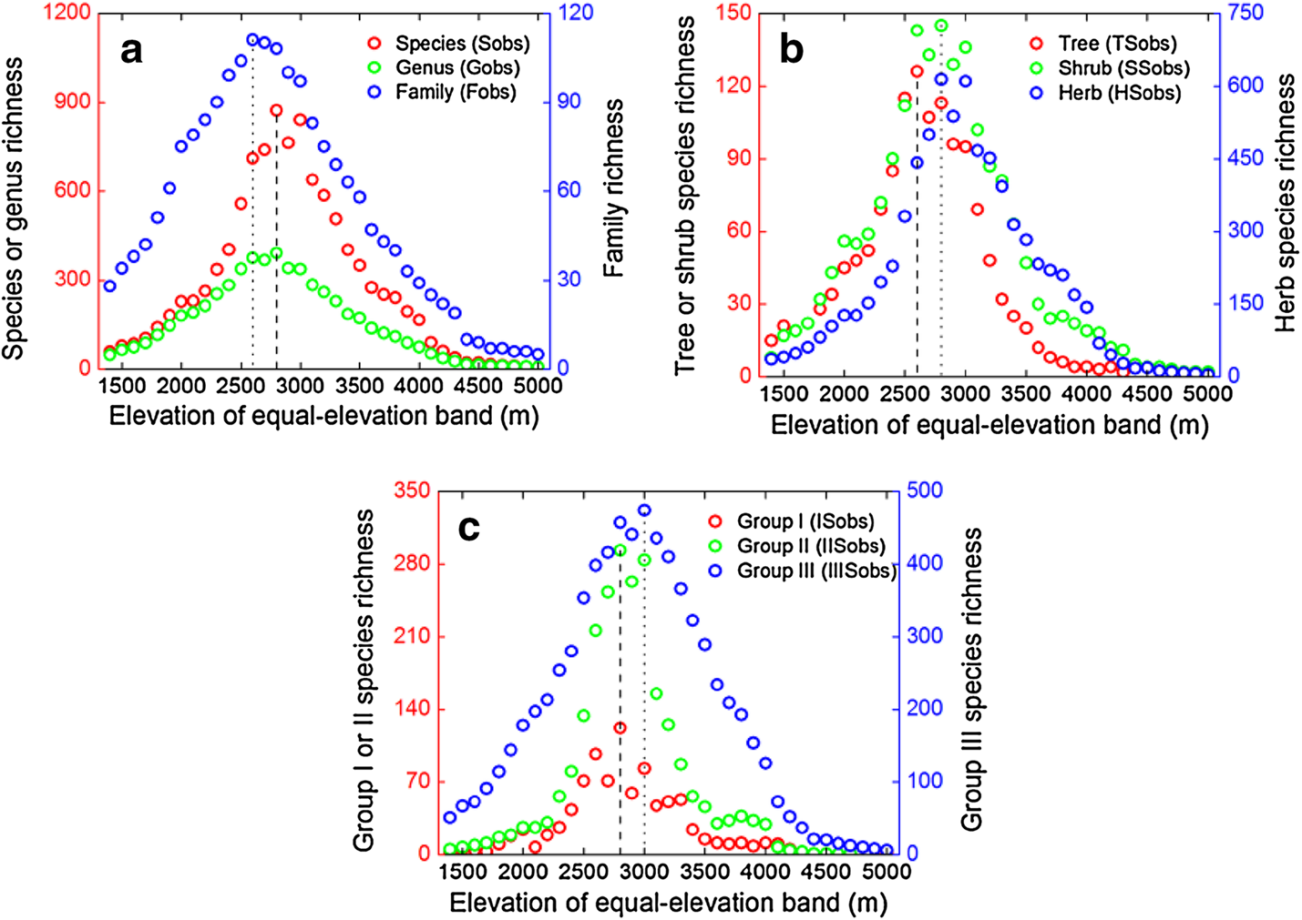
Altitudinal patterns of **a** seed plant species richness (Sobs), genus richness (Gobs), family richness (Fobs); **b** tree species richness (TSobs), shrub species richness (SSobs), herb species richness (HSobs); **c** Group I (species with elevational range size <150 m) species richness (ISobs), Group II (species with elevational range size between 150 and 500 m) species richness (IISobs), and Group III (species with elevational range size >500 m) species richness (IIISobs) along the equal-elevation gradient. The *dashed* and *dotted lines* reveal the elevations at maximum richness

Without accounting for area, Sobs, Gobs, and Hobs showed hump-shaped patterns along the equal-elevation altitudinal gradient: richness increased steeply with elevation at low elevations, and then decreased at high elevations after peaking at intermediate elevations (between 2750 and 2850 m for both species richness and genus richness, and between 2550 and 2650 m for family richness) (Fig. 5a; Table 1). TSobs, SSobs, HSobs also showed hump-shaped curves along the equal-elevation gradient, with maxima at approximately the same altitude (2550–2850 m; Fig. 5b). Moreover, ISobs, IISobs, and IIISobs also illustrated hump-shaped patterns along the equal-elevation gradient (Fig. 5c). Maximum species richness for Group I and Group II appeared at the 2750–2850 m elevation interval, whereas Group III maximum species richness occurred at 2950–3050 m.

**Table 1:** Simultaneous autoregressive models fit observed taxon richness against the first- and second-order polynomials of four variables (elevation, mean annual temperature, mean annual precipitation, and prediction from mid-domain effect)

Observed taxon richness	*n*	First-order			Second-order			
		*S*	*C*	*r* ^2^	*Q*	*S*	*C*	*r* ^2^
Elevation								
Seed plant species richness	37	−4.5 × 10^−2^	4.2 × 10^2^	0.932***	−1.3 × 10^−4^	7.5 × 10^−1^	−6.6 × 10^2^	0.939***
Seed plant genus richness	37	1.7 × 10^−3^	1.4 × 10^2^	0.972***	−4.4 × 10^−5^	2.6 × 10^−1^	−1.8 × 10^2^	0.975***
Seed plant family richness	37	1.1 × 10^−2^	1.0 × 10	0.984***	−1.2 × 10^−5^	7.9 × 10^−2^	−6.6 × 10	0.986***
Tree species richness	30	−3.8 × 10^−3^	5.3 × 10	0.931***	−2.6 × 10^−5^	1.4 × 10^−1^	−1.2 × 10^2^	0.939***
Shrub species richness	37	−5.5 × 10^−3^	6.5 × 10	0.937***	−2.2 × 10^−5^	1.3 × 10^−1^	−1.1 × 10^2^	0.944***
Herb species richness	37	−2.9 × 10^−2^	2.9 × 10^2^	0.924***	−9.5 × 10^−5^	5.8 × 10^−1^	−5.5 × 10^2^	0.931***
Group I species richness	29	7.4 × 10^−4^	2.9 × 10	0.527***	−3.6 × 10^−5^	2.0 × 10^−1^	−2.3 × 10^2^	0.651**
Group II species richness	34	−8.8 × 10^−3^	9.6 × 10	0.888***	−4.1 × 10^−5^	2.4 × 10^−1^	−2.4 × 10^2^	0.894***
Group III species richness	37	1.4 × 10^−2^	1.4 × 10^2^	0.978***	−4.6 × 10^−5^	2.9 × 10^−1^	−2.0 × 10^2^	0.980***
Mean annual temperature								
Seed plant species richness	37	1.1 × 10	1.8 × 10^2^	0.933***	−4.9	9.4 × 10	5.9	0.940***
Seed plant genus richness	37	3.7	1.1 × 10^2^	0.972***	−1.7	3.3 × 10	5.5 × 10	0.974***
Seed plant family richness	37	1.2 × 10^−1^	4.6 × 10	0.983***	−4.6 × 10^−1^	6.6	4.6 × 10	0.986***
Tree species richness	30	1.7	2.4 × 10	0.932***	−1.0	2.3 × 10	−5.8 × 10	0.939***
Shrub species richness	37	1.8	3.2 × 10	0.937***	−7.7 × 10^−1^	1.5 × 10	2.3	0.943***
Herb species richness	37	7.0	1.4 × 10^2^	0.924***	−3.8	7.1 × 10	1.6	0.933***
Group I species richness	29	6.3 × 10^−2^	3.1 × 10	0.527***	−1.2	2.6 × 10	−8.2 × 10	0.659**
Group II species richness	34	2.3	4.8 × 10	0.888***	−1.7	3.4 × 10	−4.4 × 10	0.896***
Group III species richness	37	3.2	1.6 × 10^2^	0.978***	−1.9	3.4 × 10	1.1 × 10^2^	0.980***
Mean annual precipitation								
Seed plant species richness	37	6.4 × 10^−1^	−3.3 × 10^2^	0.932***	−1.5 × 10^−2^	3.0 × 10	−1.4 × 10^4^	0.933***
Seed plant genus richness	37	1.9 × 10^−1^	−3.6 × 10	0.972***	−3.2 × 10^−3^	6.4	−3.0 × 10^3^	0.972***
Seed plant family richness	37	−5.3 × 10^−2^	9.8 × 10	0.984***	−5.7 × 10^−4^	1.1	−4.4 × 10^2^	0.984***
Tree species richness	30	6.3 × 10^−2^	−1.9 × 10	0.931***	−2.2 × 10^−3^	4.3	−2.1 × 10^3^	0.932***
Shrub species richness	37	7.7 × 10^−2^	−2.7 × 10	0.937***	−2.1 × 10^−3^	4.1	−2.0 × 10^3^	0.937***
Herb species richness	37	5.2 × 10^−1^	−3.0 × 10^2^	0.924***	−1.2 × 10^−2^	2.3 × 10	−1.1 × 10^4^	0.924***
Group I species richness	29	5.7 × 10^−1^	−5.1 × 10^2^	0.573***	−1.4 × 10^−2^	2.8 × 10	−1.4 × 10^4^	0.596***
Group II species richness	34	3.5 × 10^−1^	−2.6 × 10^2^	0.889***	−6.9 × 10^−3^	1.4 × 10	−6.7 × 10^3^	0.890***
Group III species richness	37	1.9 × 10^−1^	−1.6	0.978***	−3.8 × 10^−3^	7.6	−3.6 × 10^3^	0.979***
Mid-domain effect								
Seed plant species richness	37	6.9 × 10^−1^	1.2 × 10^2^	0.935***	6.8 × 10^−3^	−7.6 × 10^−1^	−1.5 × 10	0.942***
Seed plant genus richness	37	6.5 × 10^−1^	6.3 × 10	0.974***	4.9 × 10^−3^	1.5 × 10^−1^	1.1 × 10	0.976***
Seed plant family richness	37	5.5 × 10^−1^	2.6 × 10	0.986***	9.0 × 10^−3^	2.6 × 10^−1^	1.2 × 10	0.987***
Tree species richness	30	9.9 × 10^−1^	1.1 × 10	0.935***	2.1 × 10^−2^	4.6 × 10^−1^	2.4	0.935***
Shrub species richness	37	7.3 × 10^−1^	1.9 × 10	0.941***	2.8 × 10^−2^	−2.2 × 10^−1^	−1.5	0.943***
Herb species richness	37	7.6 × 10^−1^	7.1 × 10	0.927***	1.1 × 10^−2^	−1.1	−5.1	0.937***
Group I species richness	29	6.2 × 10^−1^	1.8 × 10	0.529***	−5.0 × 10^−1^	1.8 × 10	−1.1 × 10^2^	0.531***
Group II species richness	34	3.0 × 10^−1^	5.5 × 10	0.888***	1.7 × 10^−3^	2.2 × 10^−1^	5.4 × 10	0.888***
Group III species richness	37	6.1 × 10^−1^	9.0 × 10	0.980***	5.1 × 10^−3^	−2.9 × 10^−2^	−7.8 × 10^−1^	0.982***

Significant models are marked with asterisks *** (*p* < 0.001), ** (*p* < 0.01) and * (*p* < 0.05). The models with the lowest Akaike’s information criterion (ΔAIC > 2) are shown in the italics, and the models with ∆AIC < 2 are underlined

Group I, species with range size <150 m; Group II, species with range size between 150 and 500 m; Group III, species with range size >500 m; *n*, number of samples; *r* ^*2*^, pseudo-*R*-squared. The quadratic coefficient, slope, and constant are labeled “Q”, “S” and “C” respectively

### Effect of interpolation

**Fig. 6:**
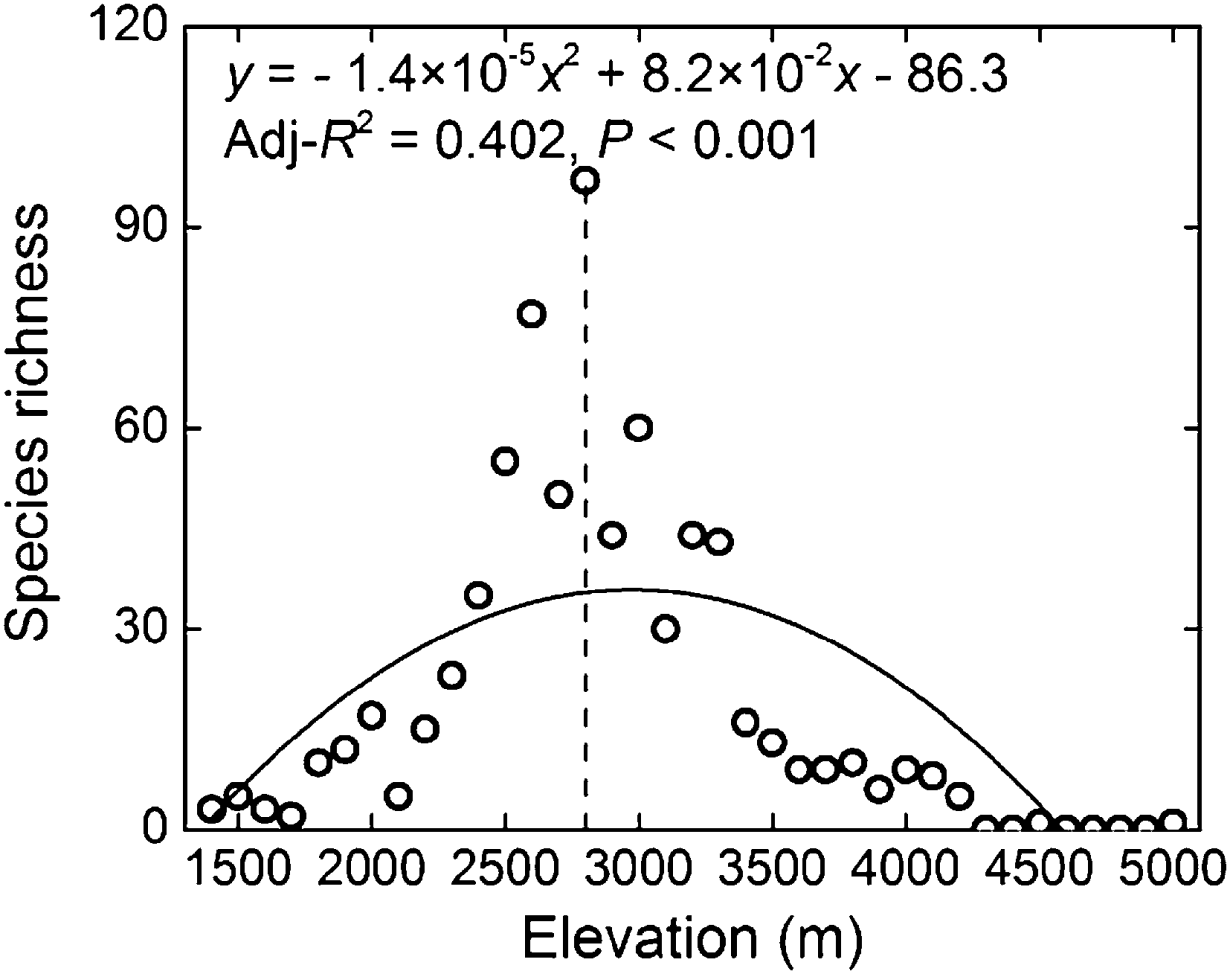
The altitudinal pattern of species richness for a plant species set (species present at only a single elevation record). The *solid line* represents the second-order polynomial fit; adj-*R* ^2^ evaluates the fitness and *p* shows the statistical significance. The *dashed line* represents the elevation at maximum species richness

According to Vetaas and Grytnes (2002), we should rule out the possibility that the interpolation approach generates an artificial hump-shaped pattern in species richness. In this study, a particular species set (717 species that have been recorded only in a single elevational band) provides an appropriate opportunity to check whether the hump-shaped species richness pattern found in Jade Dragon Snow Mountain is real. Without interpolation, this particular species set indeed showed a hump-shaped pattern in species richness with a peak at 2800 m and changes in elevation could explain 40.2 % of the variation in species richness (Fig. 6). This implies that the hump-shaped pattern for plant species in the Jade Dragon Snow Mountain may not due solely to interpolation.

### Altitudinal patterns of area-corrected taxon richness

**Fig. 7:**
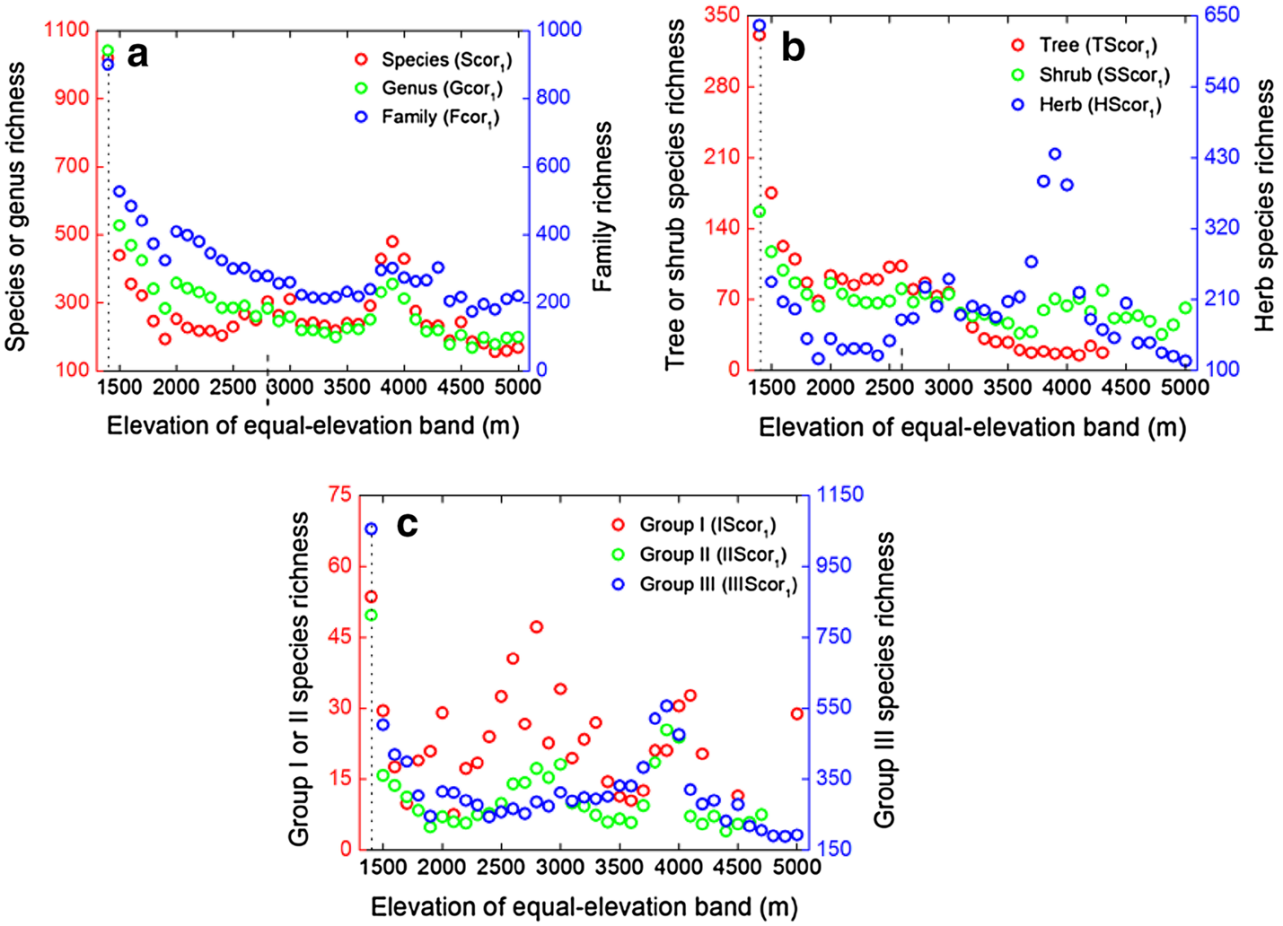
Altitudinal patterns of area-corrected **a** seed plant species richness (Scor_1_), genus richness (Gcor_1_), family richness (Fcor_1_), **b** tree species richness (TScor_1_), shrub species richness (SScor_1_), herb species richness (HScor_1_), **c** Group I species richness (IScor_1_), Group II species richness (IIScor_1_), and Group III species richness (IIIScor_1_) along the equal-elevation gradient. The *dotted-lines* demonstrate the elevations at maximum richness. For abbreviations, see Fig. 5

**Fig. 8:**
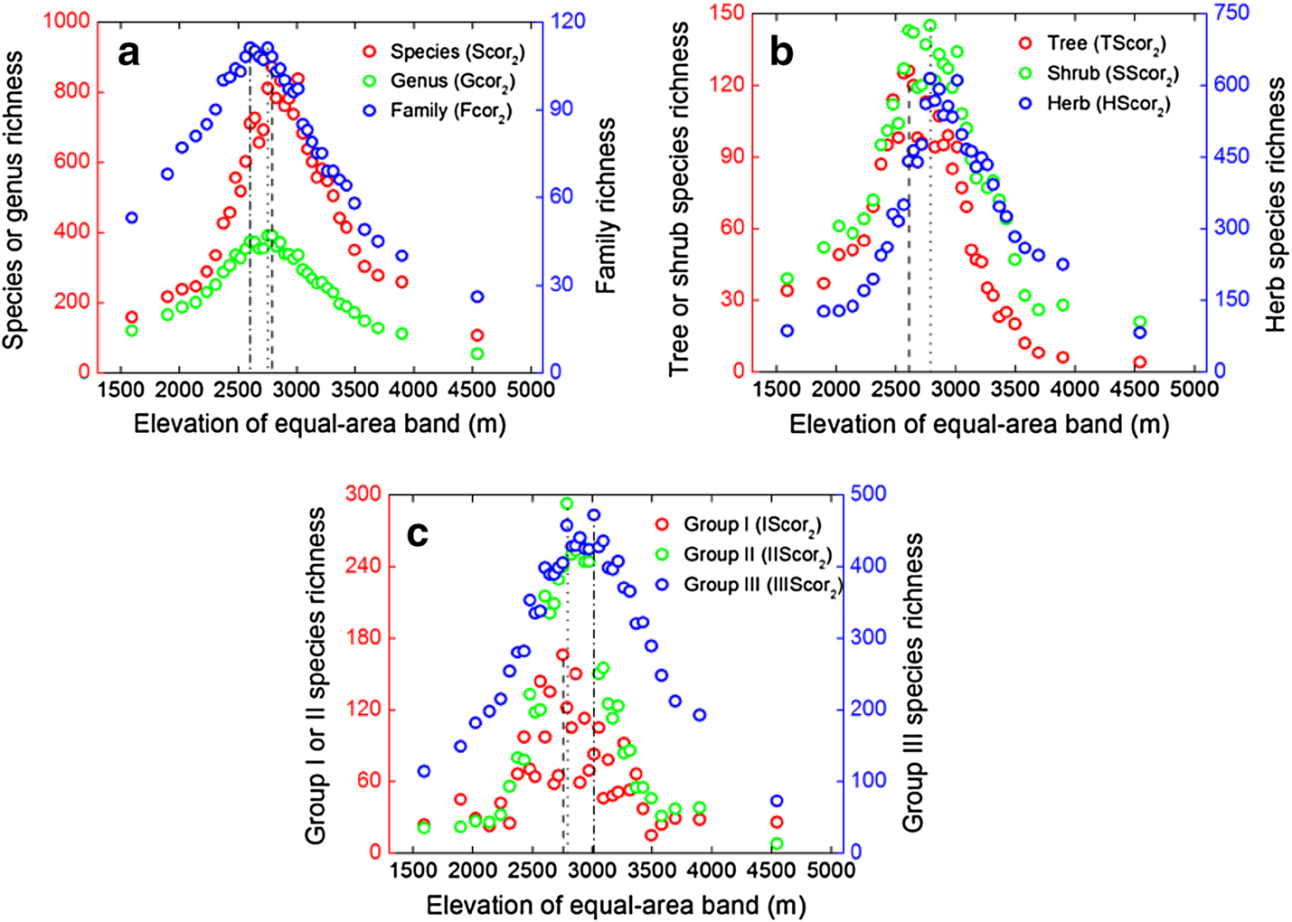
Altitudinal patterns of area-corrected **a** seed plant species richness (Scor_2_), genus richness (Gcor_2_), family richness (Fcor_2_), **b** tree species richness (TScor_2_), shrub species richness (SScor_2_), herb species richness (HScor_2_), **c** Group I species richness (IScor_2_), Group II species richness (IIScor_2_), and Group III species richness (IIIScor_2_) along the equal-area gradient. The *dashed*, *dotted*, and *dash-dotted lines* represent the elevations at maximum richness. For abbreviations, see Fig. 5

When area is accounted for using method 1, all nine TRcor_1_ variables reached their maximum richness at the lowest elevational band (roughly 1400 m), which did not match the hump-shaped pattern (Fig. 7; Table 2). Along the equal-elevation altitudinal gradient, TRcor_1_ first decreased and then oscillated, rather than showing a monotonically decreasing pattern. When accounting for area by method 2, Scor_2_, Gcor_2_, and Fcor_2_ showed hump-shaped patterns with peaks at intermediate elevations (2770.9–2807.5 m for Scor_2_, 2735.4–2807.5 m for Gcor_2_, 2586.5–2626.7 m and 2735.4–2770.9 m for Fcor_2_; Fig. 8a; Table 3). Along the equal-area altitudinal gradient, TScor_2_, SScor_2_, and HScor_2_ also showed hump-shaped curves, with maxima at nearly the same altitudes as those for taxon richness (2586.5–2626.7 m for TScor_2_, 2770.9–2807.5 m for SScor_2_ and HScor_2_; Fig. 8b). Moreover, IScor_2_, IIScor_2_, and IIIScor_2_ also had hump-shaped patterns (Fig. 8c), with maxima at 2735.4–2770.9, 2770.9–2807.5 and 2993.2–3033.2 m respectively.

**Table 2:** Simultaneous autoregressive models fit area-corrected taxon richness achieved by method 1 against the first- and second-order polynomials of four variables (elevation, mean annual temperature, mean annual precipitation, and prediction from mid-domain effect)

Area-corrected taxon richness 1	*n*	First-order			Second-order			
		*S*	*C*	*r* ^2^	*Q*	*S*	*C*	*r* ^2^
Elevation								
Seed plant species richness	37	−6.4 × 10^−2^	4.9 × 10^2^	0.307***	5.4 × 10^−5^	−4.1 × 10^−1^	9.9 × 10^2^	0.351***
Seed plant genus richness	37	−9.8 × 10^−2^	6.2 × 10^2^	0.504***	6.9 × 10^−5^	−5.3 × 10^−1^	1.2 × 10^3^	0.605*
Seed plant family richness	37	−9.6 × 10^−2^	6.2 × 10^2^	0.624***	6.2 × 10^−5^	−4.9 × 10^−1^	1.2 × 10^3^	0.727*
Tree species richness	30	−6.2 × 10^−2^	2.5 × 10^2^	0.689***	3.5 × 10^−5^	−2.6 × 10^−1^	5.1 × 10^2^	0.737***
Shrub species richness	37	−1.5 × 10^−2^	1.2 × 10^2^	0.628***	1.1 × 10^−5^	−8.5 × 10^−2^	2.1 × 10^2^	0.702***
Herb species richness	37	−2.4 × 10^−2^	2.9 × 10^2^	0.266***	2.0 × 10^−5^	−1.6 × 10^−1^	4.8 × 10^2^	0.276***
Group I species richness	29	−2.4 × 10^−3^	3.1 × 10	0.115	1.6 × 10^−6^	−1.2 × 10^−2^	4.2 × 10	0.120
Group II species richness	34	−3.1 × 10^−3^	2.1 × 10	0.218**	3.2 × 10^−6^	−2.3 × 10^−2^	4.8 × 10	0.254***
Group III species richness	37	−7.1 × 10^−2^	5.6 × 10^2^	0.369***	5.9 × 10^−5^	−4.5 × 10^−1^	1.1 × 10^3^	0.410***
Mean annual temperature								
Seed plant species richness	37	1.1 × 10	2.0 × 10^2^	0.295***	1.4	−1.3 × 10	2.5 × 10^2^	0.320***
Seed plant genus richness	37	1.7 × 10	1.5 × 10^2^	0.488***	2.2	−2.0 × 10	2.4 × 10^2^	0.567*
Seed plant family richness	37	1.7 × 10	1.6 × 10^2^	0.609***	2.0	−1.9 × 10	2.5 × 10^2^	0.695*
Tree species richness	30	1.1 × 10	−4.0 × 10	0.670***	9.8 × 10^−1^	−9.5	4.3 × 10	0.705**
Shrub species richness	37	2.6	4.5 × 10	0.619***	3.5 × 10^−1^	−3.4	5.9 × 10	0.679***
Herb species richness	37	3.6	1.8 × 10^2^	0.261***	2.8 × 10^−1^	−1.1	1.9 × 10^2^	0.263***
Group I species richness	29	3.9 × 10^−1^	2.0 × 10	0.111	2.3 × 10^−2^	−1.0 × 10^−1^	2.2 × 10	0.112
Group II species richness	34	5.0 × 10^−1^	7.1	0.209**	7.4 × 10^−2^	−8.8 × 10^−1^	1.1 × 10	0.227**
Group III species richness	37	1.2 × 10	2.4 × 10^2^	0.358***	1.5	−1.3 × 10	2.9 × 10^2^	0.380***
Mean annual precipitation								
Seed plant species richness	37	−2.5	2.7 × 10^3^	0.295***	2.8 × 10^−2^	−5.5 × 10	2.8 × 10^4^	0.299**
Seed plant genus richness	37	−1.6	1.9 × 10^3^	0.379***	2.7 × 10^−2^	−5.3 × 10	2.6 × 10^4^	0.382***
Seed plant family richness	37	−1.5	1.7 × 10^3^	0.500***	2.6 × 10^−2^	−5.1 × 10	2.5 × 10^4^	0.504***
Tree species richness	30	−2.9 × 10^−1^	3.6 × 10^2^	0.533***	−8.4 × 10^−4^	1.3	−4.1 × 10^2^	0.533***
Shrub species richness	37	−2.7 × 10^−1^	3.2 × 10^2^	0.556***	2.1 × 10^−4^	−6.7 × 10^−1^	5.2 × 10^2^	0.556***
Herb species richness	37	−2.1	2.3 × 10^3^	0.341**	6.1 × 10^−3^	−1.4 × 10	7.9 × 10^3^	0.342**
Group I species richness	29	4.2 × 10^−2^	−1.6 × 10	0.102	−1.8 × 10^−3^	3.5	−1.6 × 10^3^	0.106
Group II species richness	34	−8.3 × 10^−2^	9.1 × 10	0.195**	−7.7 × 10^−4^	1.4	−6.2 × 10^2^	0.196**
Group III species richness	37	−3.0	3.2 × 10^3^	0.372***	3.4 × 10^−2^	−6.8 × 10	3.4 × 10^4^	0.378***
Mid-domain effect								
Seed plant species richness	37	−9.0 × 10^−1^	5.2 × 10^2^	0.317***	1.2 × 10^−2^	−5.6	8.1 × 10^2^	0.493***
Seed plant genus richness	37	−1.5	5.2 × 10^2^	0.452***	2.7 × 10^−2^	−8.1	7.9 × 10^2^	0.554***
Seed plant family richness	37	−4.5	5.5 × 10^2^	0.571***	2.0 × 10^−1^	−2.1 × 10	7.9 × 10^2^	0.645***
Tree species richness	30	−1.9 × 10	7.2 × 10^2^	0.857***	4.8 × 10^−1^	−2.9 × 10	4.8 × 10^2^	0.954***
Shrub species richness	37	−1.3	1.2 × 10^2^	0.647***	3.8 × 10^−2^	−3.4	1.3 × 10^2^	0.697***
Herb species richness	37	−5.0 × 10^−1^	3.0 × 10^2^	0.274***	1.7 × 10^−2^	−5.1	5.1 × 10^2^	0.451***
Group I species richness	29	−2.5	7.9 × 10	0.349	−4.2 × 10^−1^	1.2 × 10	−3.1 × 10	0.356
Group II species richness	34	−8.6 × 10^−1^	5.8 × 10	0.580***	3.1 × 10^−2^	−2.9	7.5 × 10	0.767***
Group III species richness	37	−8.0 × 10^−1^	4.8 × 10^2^	0.344***	2.0 × 10^−2^	−6.6	7.5 × 10^2^	0.470***

Significant models are marked with asterisks *** (*p* < 0.001), ** (*p* < 0.01), and * (*p* < 0.05). The models with the lowest Akaike’s information criterion (ΔAIC > 2) are shown in the italics, and the models with ∆AIC < 2 are underlined. For abbreviations, see Table 1

**Table 3:** Simultaneous autoregressive models fit area-corrected taxon richness achieved by method 2 against the first- and second-order polynomials of four variables (elevation, mean annual temperature, mean annual precipitation and prediction from mid-domain effect)

Area-corrected taxon richness 2	*n*	First-order			Second-order			
		*S*	*C*	*r* ^2^	*Q*	*S*	*C*	*r* ^2^
Elevation								
Seed plant species richness	37	−4.0 × 10^−3^	2.2 × 10^2^	0.893***	−9.8 × 10^−5^	5.9 × 10^−1^	−5.4 × 10^2^	0.920***
Seed plant genus richness	37	−2.8 × 10^−2^	2.1 × 10^2^	0.926***	−3.9 × 10^−5^	2.1 × 10^−1^	−9.5 × 10	0.952***
Seed plant family richness	37	−1.3 × 10^−2^	8.9 × 10	0.920***	−1.0 × 10^−5^	4.7 × 10^−2^	1.2 × 10	0.946***
Tree species richness	37	−1.4 × 10^−2^	6.6 × 10	0.904***	−2.0 × 10^−6^	−2.6 × 10^−3^	5.1 × 10	0.905***
Shrub species richness	37	−1.1 × 10^−2^	7.0 × 10	0.870***	−6.6 × 10^−6^	2.9 × 10^−2^	1.9 × 10	0.874***
Herb species richness	37	2.1 × 10^−2^	8.8 × 10	0.884***	−9.1 × 10^−5^	5.7 × 10^−1^	−6.2 × 10^2^	0.927***
Group I species richness	37	−3.6 × 10^−3^	6.0 × 10	0.231***	−3.1 × 10^−5^	1.8 × 10^−1^	−1.9 × 10^2^	0.346*
Group II species richness	37	−2.3 × 10^−4^	2.8 × 10	0.853***	−2.1 × 10^−5^	1.2 × 10^−1^	−1.3 × 10^2^	0.859***
Group III species richness	37	−2.3 × 10^−3^	1.6 × 10^2^	0.862***	−7.7 × 10^−5^	4.6 × 10^−1^	−4.3 × 10^2^	0.933***
Mean annual temperature								
Seed plant species richness	37	5.0 × 10^−1^	2.1 × 10^2^	0.893***	−4.5	9.1 × 10	−7.5 × 10	0.921***
Seed plant genus richness	37	5.2	7.5 × 10	0.926***	−1.8	4.1 × 10	−3.8 × 10	0.954***
Seed plant family richness	37	2.5	2.7 × 10	0.922***	−4.6 × 10^−1^	1.2 × 10	−1.9	0.951***
Tree species richness	37	2.8	−4.8	0.906***	−1.3 × 10^−1^	5.3	−1.3 × 10	0.907***
Shrub species richness	37	2.1	1.7 × 10	0.871***	−3.3 × 10^−1^	8.6	−3.0	0.876***
Herb species richness	37	−4.3	1.9 × 10^2^	0.885***	−4.2	7.9 × 10	−6.4 × 10	0.930***
Group I species richness	37	7.6 × 10^−1^	4.2 × 10	0.232***	−1.3	2.7 × 10	−6.0 × 10	0.368*
Group II species richness	37	−5.3 × 10^−3^	2.7 × 10	0.853***	−1.0	2.1 × 10	−3.7 × 10	0.860***
Group III species richness	37	2.3 × 10^−1^	1.5 × 10^2^	0.862***	−3.6	7.1 × 10	−6.8 × 10	0.937***
Mean annual precipitation								
Seed plant species richness	37	3.6 × 10^−1^	−1.3 × 10^2^	0.893***	4.4 × 10^−2^	−8.5 × 10	4.1 × 10^4^	0.894***
Seed plant genus richness	37	1.3	−1.1 × 10^3^	0.933***	−2.2 × 10^−2^	4.3 × 10	−2.1 × 10^4^	0.934***
Seed plant family richness	37	5.6 × 10^−1^	−4.8 × 10^2^	0.932***	−1.9 × 10^−2^	3.7 × 10	−1.8 × 10^4^	0.945***
Tree species richness	37	5.7 × 10^−1^	−5.2 × 10^2^	0.898***	−3.9 × 10^−2^	7.6 × 10	−3.7 × 10^4^	0.918***
Shrub species richness	37	5.1 × 10^−1^	−4.5 × 10^2^	0.880***	−3.0 × 10^−2^	5.9 × 10	−2.9 × 10^4^	0.892***
Herb species richness	37	−6.9 × 10^−1^	8.1 × 10^2^	0.881***	1.1 × 10^−1^	−2.1 × 10^2^	1.0 × 10^5^	0.890***
Group I species richness	37	6.7 × 10^−1^	−5.9 × 10^2^	0.279**	−5.5 × 10^−2^	1.1 × 10^2^	−5.2 × 10^4^	*0.383*
Group II species richness	37	4.5 × 10^−2^	−1.6 × 10	0.853***	−1.5 × 10^−2^	3.0 × 10	−1.4 × 10^4^	0.853***
Group III species richness	37	2.2 × 10^−1^	−5.1 × 10	0.863***	3.2 × 10^−2^	−6.2 × 10	3.0 × 10^4^	0.865***
Mid-domain effect								
Seed plant species richness	37	7.3 × 10^−1^	3.7 × 10	0.909***	3.9 × 10^−3^	−1.1	2.3 × 10^2^	0.911***
Seed plant genus richness	37	1.1 × 10^−1^	1.1 × 10^2^	0.890***	−3.2 × 10^−3^	9.5 × 10^−1^	6.7 × 10	0.892***
Seed plant family richness	37	−1.2 × 10^−1^	5.8 × 10	0.801***	−1.6 × 10^−2^	1.4	2.7 × 10	0.810***
Tree species richness	37	−8.8 × 10^−1^	5.0 × 10	0.858***	−6.1 × 10^−2^	2.6	4.6	0.861***
Shrub species richness	37	−2.2 × 10^−1^	4.7 × 10	0.839***	−3.6 × 10^−2^	2.7	−5.0	0.844***
Herb species richness	37	1.2	−5.3 × 10	0.923***	1.0 × 10^−2^	−2.1	1.9 × 10^2^	0.930***
Group I species richness	37	5.8 × 10	−1.3 × 10^3^	0.271***	9.6 × 10	−4.3 × 10^3^	4.9 × 10^4^	0.279***
Group II species richness	37	4.6 × 10^−1^	2.6	0.853***	2.5	−2.4 × 10^2^	5.5 × 10^3^	0.857***
Group III species richness	37	6.4 × 10^−1^	5.1 × 10	0.909***	2.7 × 10^−3^	−2.3 × 10^−1^	1.1 × 10^2^	0.911***

Significant models are marked with asterisks *** (*p* < 0.001), ** (*p* < 0.01), and * (*p* < 0.05). The models with the lowest Akaike’s information criterion (ΔAIC > 2) are shown in the italics type, and the models with ∆AIC < 2 are underlined. For abbreviations, see Table 1

### Roles of MAT, MAP, and MDE

**Fig. 9:**
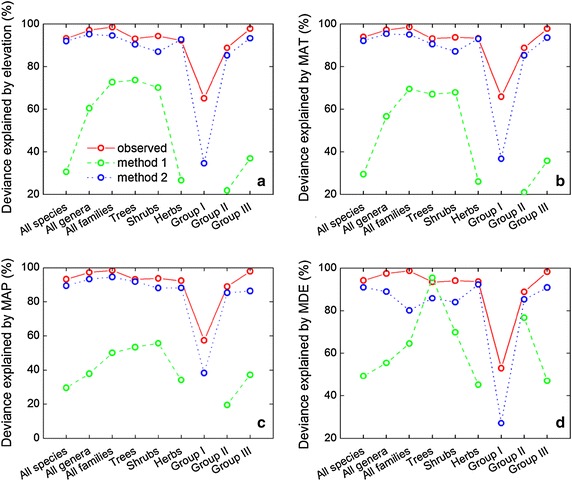
Simultaneous autoregressive models reveal the deviance explained in observed taxon richness, area-corrected taxon richness achieved by method 1 and 2 by **a** elevation, **b** mean annual temperature (MAT), **c** mean annual precipitation (MAP), and **d** prediction from mid-domain effect (MDE). For abbreviations, see Fig. 5

SAR showed significant correlations between TRobs and each explanatory variable: MAT, MAP, and MDE (Table 1). MAT accounted for 65.9–98.6 % of the variation in TRobs, MAP was responsible for 57.3–98.4 %, and MDE explained 52.9–98.6 % (Table 1; Additional file 1: Figure S4). The proportions of variation in TRobs explained by MAT, MAP, and MDE were similar for each plant group. After eliminating the area effect using method 1, significant correlations were apparent between TRcor_1_ and each explanatory variable (MAT, MAP, and MDE), except for IScor_1_ (Table 2). MAT accounted for 20.9–69.5 % of the variation in TRcor_1_, MAP explained 19.5–55.6 % of the variance, and MDE was responsible for 45.1–95.4 % (Table 2; Additional file 1: Figure S5). Generally, MDE explained more variation in TRcor_1_ than MAT and MAP. When accounting for the area effect using method 2, MAT, MAP and MDE all had significant influences on TRcor_2_ for the nine plant groups (Table 3). MAT was responsible for 36.8–95.4 % of the variance in TRcor_2_, MAP explained 38.3–94.5 % and MDE accounted for 27.1–92.3 % (Table 3; Additional file 1: Figure S6). Generally, MAT, MAP, and MDE explained a smaller proportion of the variance in IScor_2_, compared with other plant groups. For all nine plant groups, elevation, MAT, and MAP were responsible for more of the variation in TRobs than in TRcor_1_ and TRcor_2_ (Fig. 9a–c). MDE also accounted for a larger proportion of the variance in TRobs as compared with TRcor_1_ and TRcor_2_, except for trees (Fig. 9d).

## Discussion

The altitudinal patterns of plant diversity and the relative importances of hidden determinants have been a controversial issue for ecological research (Sanders 2002, Wang et al. 2007a, b). As Rahbek (1995, 2005) pointed out, a mid-elevation peak in species richness is the most common pattern in mountain regions. Along the large-scale altitudinal gradient nearest to the equator in the Northern Hemisphere (i.e., the Jade Dragon Snow Mountain), we indeed found that all measures of observed taxon richness (i.e., TRobs: Sobs, Gobs, Fobs, TSobs, SSobs, HSobs, ISobs, IISobs, and IIISobs) displayed the hump-shaped patterns (Fig. 5). For all plant groups considered in this study, the taxon richness peaked at the lower elevations, which is consistent with previous reports (VanDerWal et al. 2008).

Once we excluded the effect of the interpolation method in producing the hump-shaped patterns in taxon richness as suggested by Vetaas and Grytnes (2002), we could explicitly assess the effect of abiotic factors on the patterns. Variations in taxon richness are rarely wholly due to a single factor (Oommen and Shanker 2005). Area has a strong influence on the pattern of species richness at all scales (Whittaker et al. 2001). Larger areas contain more individuals and thus more species (‘passive-sampling hypothesis’, Connor and McCoy 1979). The analyses on the Jade Dragon Snow Mountain also yielded further evidence for the significant influence of area on these patterns. A power-law species–area relationship explained a good deal (≥53.1 %) of the variance in TRobs (Additional file 1: Table S1; Figures S1–S3). While accounting for area, TRcor_2_ still displayed hump-shaped patterns (Fig. 8; Table 3). This implies that area alone is not fully responsible for the hump-shaped patterns seen in TRobs. This is consistent with results for palms in New Guinea (Bachman et al. 2004) and seed plants in the Gaoligong Mountains of China (Wang et al. 2007a, b). However, TRcor_1_ did not show hump-shaped patterns and elevation accounted for a lower proportion of the variation in TRcor_1_ than in TRobs (Figs. 7, 9). These also confirmed the importance of area in shaping altitudinal patterns of plant diversity. Moreover, the percentage of variance explained by elevation was larger in TRcor_2_ than in TRcor_1_. This revealed that method 1 partly removed the effect of elevation along with eliminating the influence of area. However, method 2 retained the effect of elevation to the greatest degree.

Moderate temperature conditions in the middle of altitude gradients can explain the hump-shaped pattern of biodiversity (Kluge et al. 2006). As expected, TRobs, TRcor_1_, and TRcor_2_ all showed hump-shaped patterns with MAT, independent of plant groups (Tables 1, 2, 3; Additional file 1: Figures S4–S6). MAT showed a strong monotonic decreasing relationship with increasing elevation (Figs. 2b, 4b), the simple Pearson’s correlation coefficients between MAT and elevation along the equal-elevation and equal-area gradients were both −0.999 (*p* < 0.001). MAT and elevation thus explained a similar proportion of variance in all three types of taxon richness (Tables 1, 2, 3). MAT demonstrated less of the variance in area-corrected taxon richness than in observed taxon richness, which indicates collinearity between area and MAT. The simple Pearson’s correlation coefficient between area and MAT along the equal-elevation gradient was 0.334 (*p* < 0.05). Moreover, MAT explained a larger percentage of the variation in TRcor_2_ than in TRcor_1_, showing that method 1 removed more MAT effect than did method 2. This also indicates that method 2 retains the explanatory power of MAT to a great degree while eliminating the influence of area. MAP explained a greater proportion of the variance in TRobs than in TRcor_1_ and TRcor_2_, which indicates collinearity between area and MAP. The simple Pearson’s correlation coefficient between area and MAP along the equal-elevation gradient was 0.537 (*p* < 0.01). However, MAP accounted for a larger part of the variation in TRcor_2_ than in TRcor_1_. This demonstrates that method 2 is reasonable and shows the effect of MAP more clearly than method 1.

Altitudinal gradients in taxon richness have usually been attributed to a linear relationship with MDE predictions (Colwell and Lees 2000; Colwell et al. 2004), which is confirmed by our findings (Table 1; Additional file 1: Fig. S4). While accounting for the area effect, MDE accounts for less of the variation in area-corrected taxon richness compared with observed taxon richness, revealing collinearity between area and MDE. This is inconsistent with the case of palms in New Guinea (Bachman et al. 2004). Bachman et al. clarified that MDE predictions conditionally explain up to 98 % of the variance in palm taxon richness after removing the effect of area, since in New Guinea both area and observed taxon richness of palms along the equal-elevation gradient decrease monotonically with elevation (Bachman et al. 2004). However, taxon richness along the equal-area gradient showed a hump-shaped pattern, which was more consistent with the changes in MDE with elevation. Thus an area correction approach increased the explanatory power of MDE for palms in New Guinea. However, on the Jade Dragon Snow Mountain, observed taxon richness and simulated taxon richness predicted by MDE all show hump-shaped patterns along the equal-elevation altitudinal gradient. After removing the area effect by method 2, i.e., along the equal-area altitudinal gradient, both area-corrected taxon richness and simulated taxon richness evaluated by MDE still exhibit hump-shaped patterns. The strength of the relationship between TRcor_2_ and MDE is weakened when the area effect is eliminated. Except for trees, the prediction from MDE accounted for a larger part of the variation in TRcor_2_ than in TRcor_1_. This indicates that method 2 retains the influence of MDE to the maximum large extent as well as eliminating the area effect.

For patterns of plant diversity along the altitudinal gradient on the Jade Dragon Snow Mountain, our analysis implies that method 2 performs better than method 1 in terms of preserving the significant effects of MAT, MAP, and MDE. There is no doubt that the species–area relationship is one of ecology’s few laws (Rosenzweig 1995; Rahbek 1997; McCain 2007). Thus method 1, based on this law, should perform well in accounting for the area effect, and this is indeed confirmed by our results (Fig. 9; Tables 1, 2, 3). In this paper, we not only appreciate the essential contribution of method 1 to community ecology, but also highlight the significant role of method 2 in macroecological studies conducted in mountainous regions.

## Conclusions

In this study, we investigated the altitudinal patterns of taxon richness for seed plants on the Jade Dragon Snow Mountain, southwestern China. We evaluated the effect of area on taxon richness patterns (TRobs) and invoked two methods to calculate area-corrected taxon richness (TRcor_1_ and TRcor_2_). We calculated the number of species in the smallest sampling area based on a power-law species–area relationship implemented in method 1. According to method 2, we counted the number of taxa along the equal-area altitudinal gradient. We assessed the influences of MAT, MAP, and MDE on taxon richness before and after eliminating area effect. Our results reveal that both TRobs and TRcor_2_ show hump-shaped patterns along the altitudinal gradient, while TRcor_1_ dose not. Elevation, MAT, MAP, and MDE explain a smaller proportion of the variance in area-corrected taxon richness than in observed taxon richness. Moreover, they were all responsible for a larger percentage of the variance in TRcor_2_ than TRcor_1_. These findings indicate that area effects should be taken into consideration when assessing the influences of other abiotic factors on taxon richness patterns along altitudinal gradients. Method 2 performs better in controlling area effect than method 1 for seed plants on the Jade Dragon Snow Mountain, in terms of preserving the significant effects of abiotic factors. We not only strongly acknowledge the significance of method 1 in community ecology but also highlight the remarkable contribution of method 2 in eliminating area effects and delineating the explanatory power of abiotic factors. Therefore, we appeal for more application of method 2 in macroecological studies in mountainous regions.

## Abbreviations

Groups I: species with elevational range size <150 m
Groups II: species with elevational range size between 150 and 500 m
Groups III: species with elevational range size >500 m
MAT: mean annual temperature
MAP: mean annual precipitation
MDE: mid-domain effect

### Within the equal-elevation altitudinal band

TRobs: number of taxa
Sobs: number of species
Gobs: number of genera
Fobs: number of families
TSobs: number of tree species
SSobs: number of shrub species
HSobs: number of herbaceous species
ISobs: number of Groups I species
IISobs: number of Groups II species
IIISobs: number of Groups III species

### Based on a power-law species–area relationship

TRcor_1_: number of taxa
Scor_1_: number of species
Gcor_1_: number of genera
Fcor_1_: number of families
TScor_1_: number of tree species
SScor_1_: number of shrub species
HScor_1_: number of herbaceous species
IScor_1_: number of Groups I species
IIScor_1_: number of Groups II species
IIIScor_1_: number of Groups III species

### Within the equal-area altitudinal band

TRcor_2_: number of taxa
Scor_2_: number of species
Gcor_2_: number of genera
Fcor_2_: number of families
TScor_2_: number of tree species
SScor_2_: number of shrub species
HScor_2_: number of herbaceous species
IScor_2_: number of Groups I species
IIScor_2_: number of Groups II species
IIIScor_2_: number of Groups III species

## Additional file

10.1186/s40064-016-3052-1 Species–area relationships; relationships between observed taxon richness and factors; relationships between area-corrected taxon richness achieved by method 1 and factors;​ relationships between area-corrected taxon richness achieved by method 2 and factors;​ correlograms of ordinary least squares and simultaneous autoregressive model residuals.
