# Supplementary material for: Altitudinal patterns of plant diversity on the Jade Dragon Snow Mountain, southwestern China
Source: Springerplus. 2016 Sep 15;5(1):1566. doi: 10.1186/s40064-016-3052-1 (PMC5023648; doi:10.1186/s40064-016-3052-1)
Supplement: Supplementary file 1 — 10.1186/s40064-016-3052-1 Species–area relationships; relationships between observed taxon richness and factors; relationships between area-corrected taxon richness achieved by method 1 and factors;​ relationships between area-corrected taxon richness achieved by method 2 and factors;​ correlograms of ordinary least squares and simultaneous autoregressive model residuals. [file 40064_2016_3052_MOESM1_ESM.docx]

**
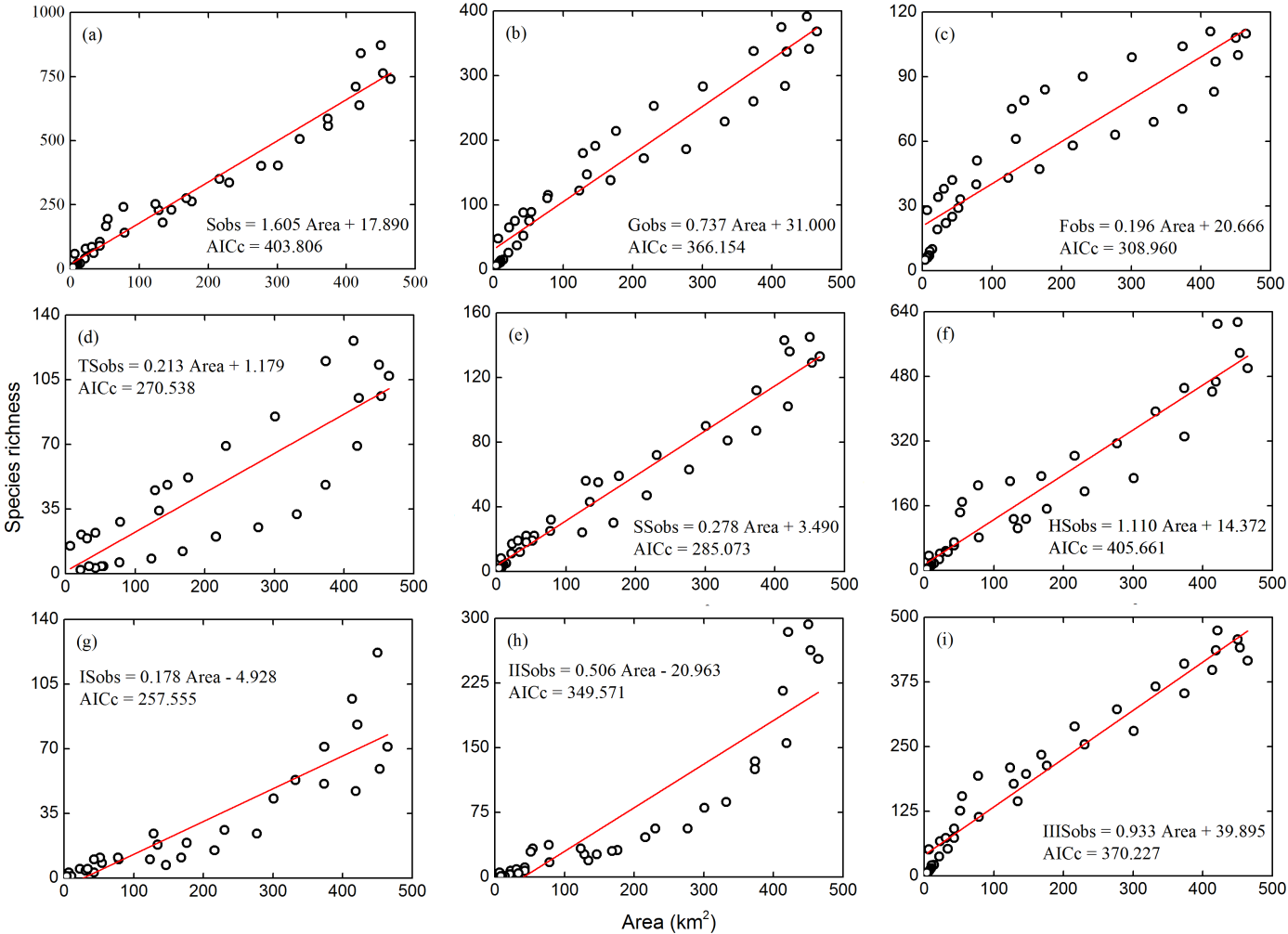
**

**Figure S1** Untransformed species–area relationships (species richness versus area) along the equal-elevation altitudinal gradient for (a) seed species richness (Sobs), (b) genus richness (Gobs), (c) family richness (Fobs), (d) tree species richness (TSobs), (e) shrub species richness (SSobs), (f) herb species richness (HSobs), (g) Group I (species with elevational range size <150 m) species richness (ISobs), (h) Group II (species with elevational range size between 150 m and 500 m) species richness (IISobs), (i) and Group III (species with elevational range size >500 m) species richness (IIISobs). Functions inside each figure are results of ordinary least squares linear regressions and values are Akaike’s information criterion corrected for small sample size (AIC_C_).

**
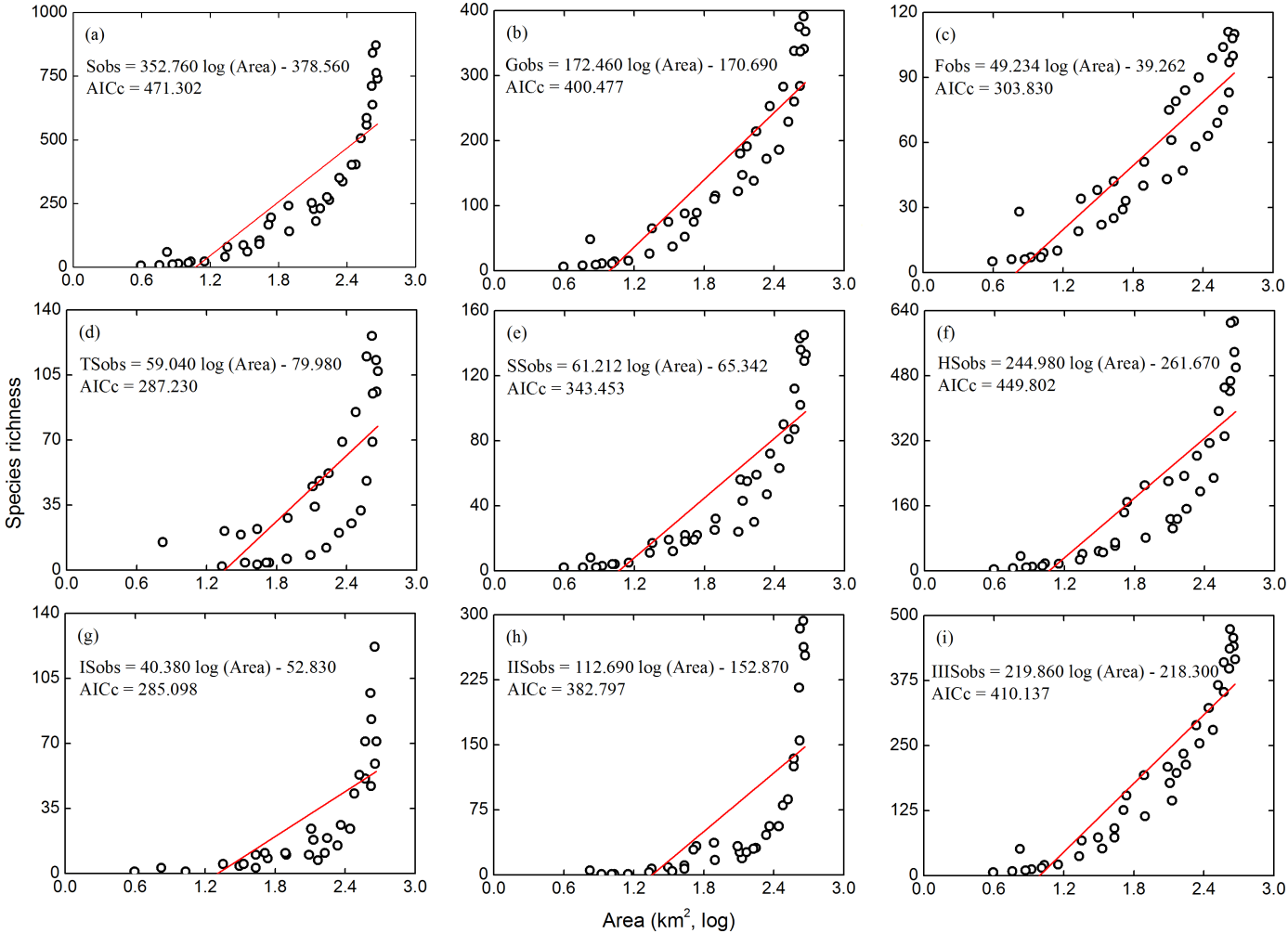
**

**Figure S2** Semi-log transformed species–area relationships (species richness versus log area) along the equal-elevation altitudinal gradient for nine plant groups. Functions inside each figure are results of ordinary least squares linear regressions and values are Akaike’s information criterion corrected for small sample size (AIC_C_). For abbreviations, see Figure S1.

**
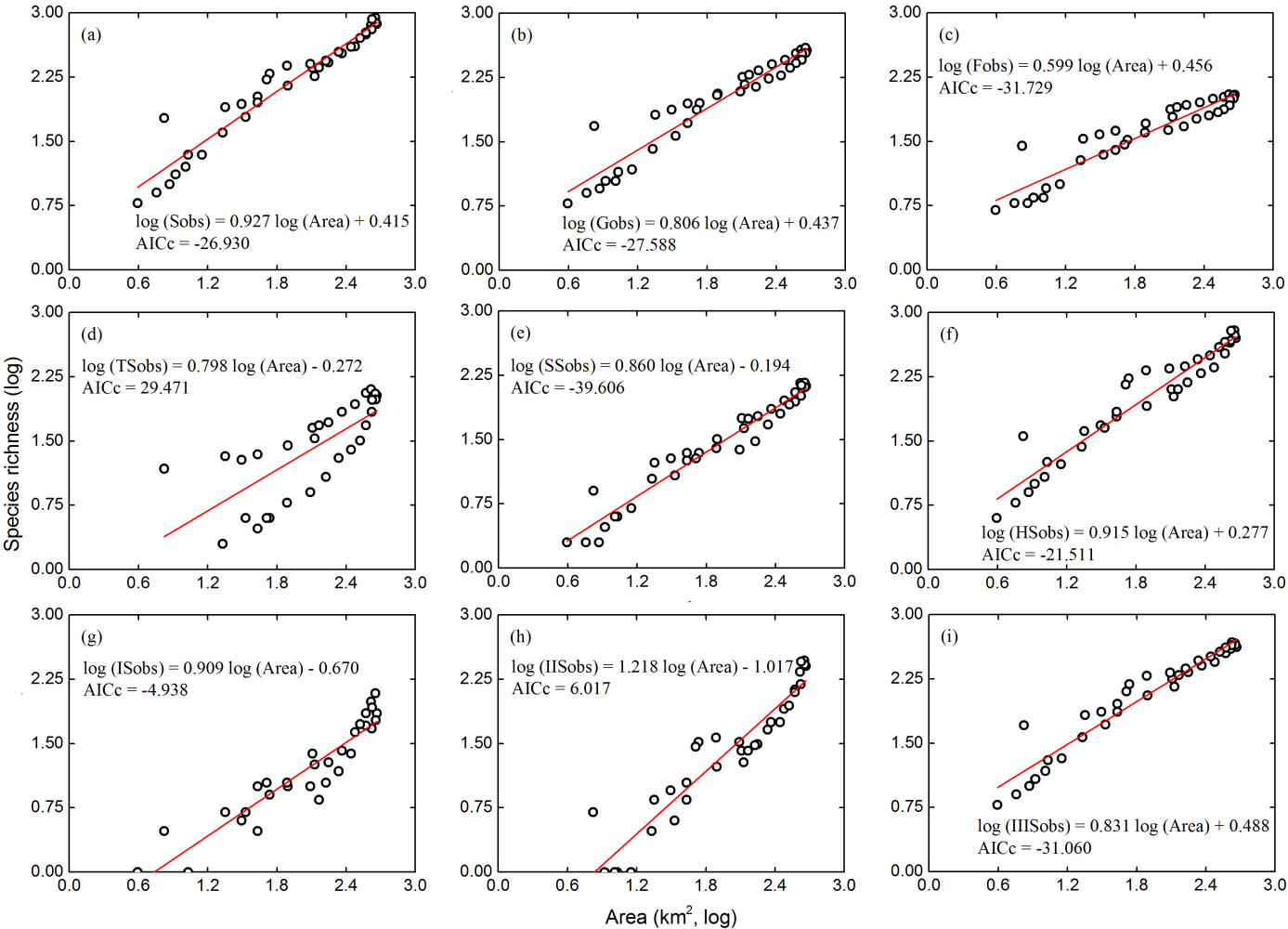
**

**Figure S3** Log–log transformed species–area relationships (log species richness versus log area) along the equal-elevation altitudinal gradient for nine plant groups. Functions inside each figure are results of ordinary least squares linear regressions and values are Akaike’s information criterion corrected for small sample size (AIC_C_). For abbreviations, see Figure S1.

**
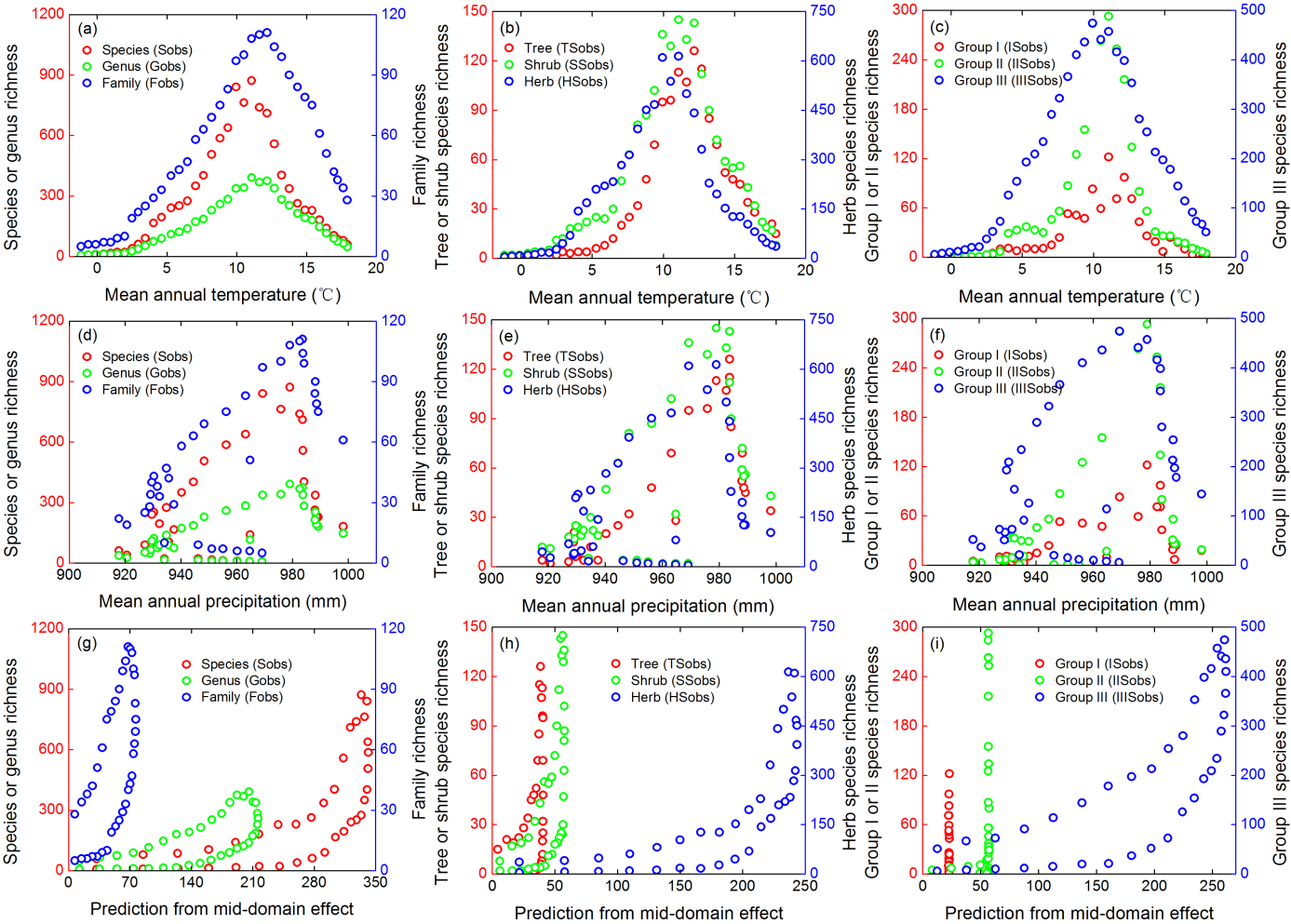
**

**Figure S4** Relationships between observed taxon richness and (a)–(c) mean annual temperature, (d)–(f) mean annual precipitation, and (g)–(i) prediction from mid-domain effect. For abbreviations, see Figure S1.


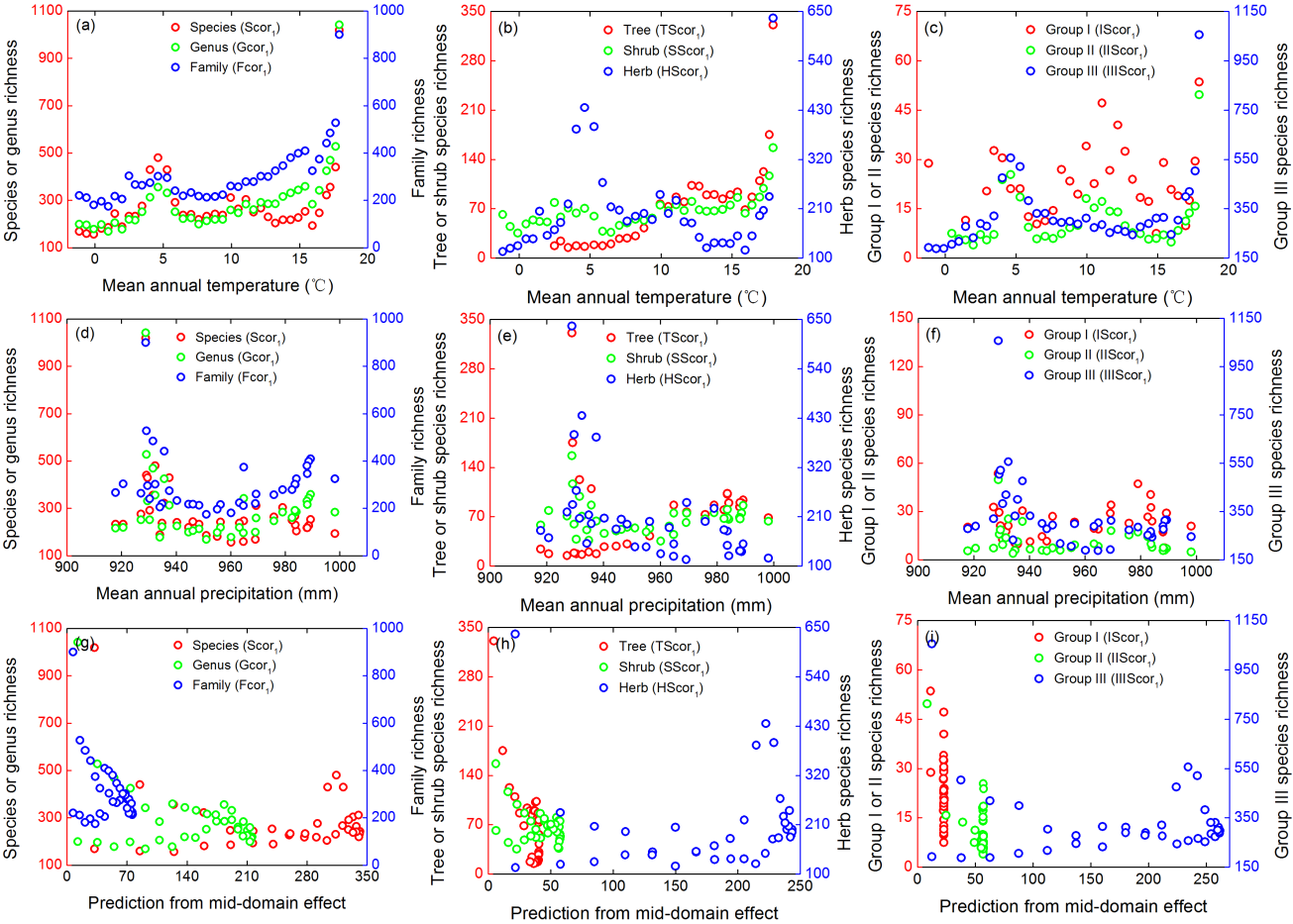


**Figure S5** Relationships between area-corrected taxon richness achieved by method 1 and (a)–(c) mean annual temperature, (d)–(f) mean annual precipitation, and (g)–(i) prediction from mid-domain effect. Abbreviations are Scor_1_, seed plant species richness; Gcor_1_, seed plant genus richness; Fcor_1_, seed plant family richness; TScor_1_, tree species richness; SScor_1_, shrub species richness; HScor_1_, herb species richness; IScor_1_, Group I (species with elevational range size <150 m) species richness; IIScor_1_, Group II (species with elevational range size between 150 m and 500 m) species richness; and IIIScor_1_, Group III (species with elevational range size >500 m) species richness.

**
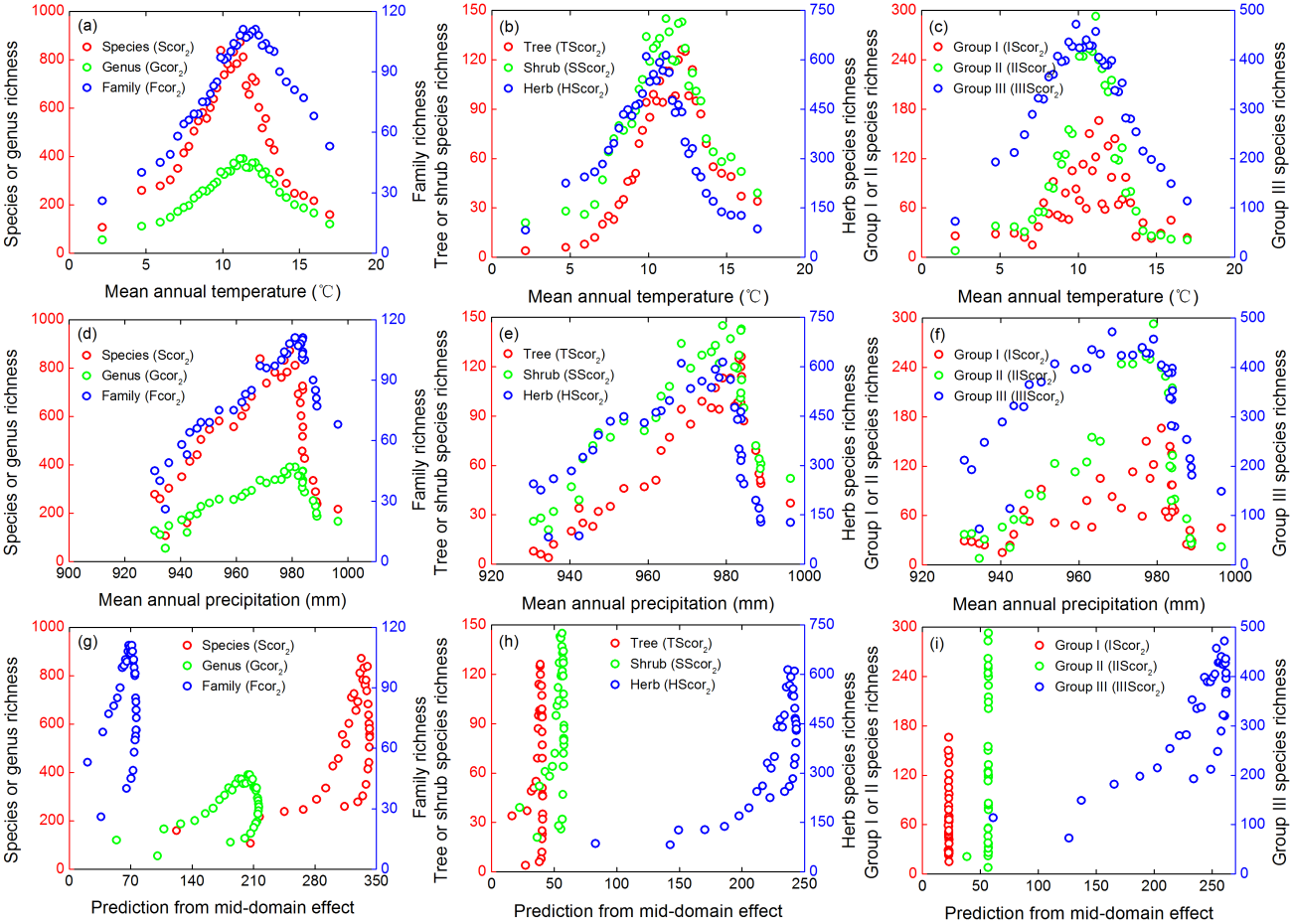
**

**Figure S6** Relationships between area-corrected taxon richness achieved by method 2 and (a)–(c) mean annual temperature, (d)–(f) mean annual precipitation, and (g)–(i) prediction from mid-domain effect. Abbreviations are Scor_2_, seed plant species richness; Gcor_2_, seed plant genus richness; Fcor_2_, seed plant family richness; TScor_2_, tree species richness; SScor_2_, shrub species richness; HScor_2_, herb species richness; IScor_2_, Group I species richness; IIScor_2_, Group II species richness; and IIIScor_2_, Group III species richness. For other abbreviations, see Figure S5.

**
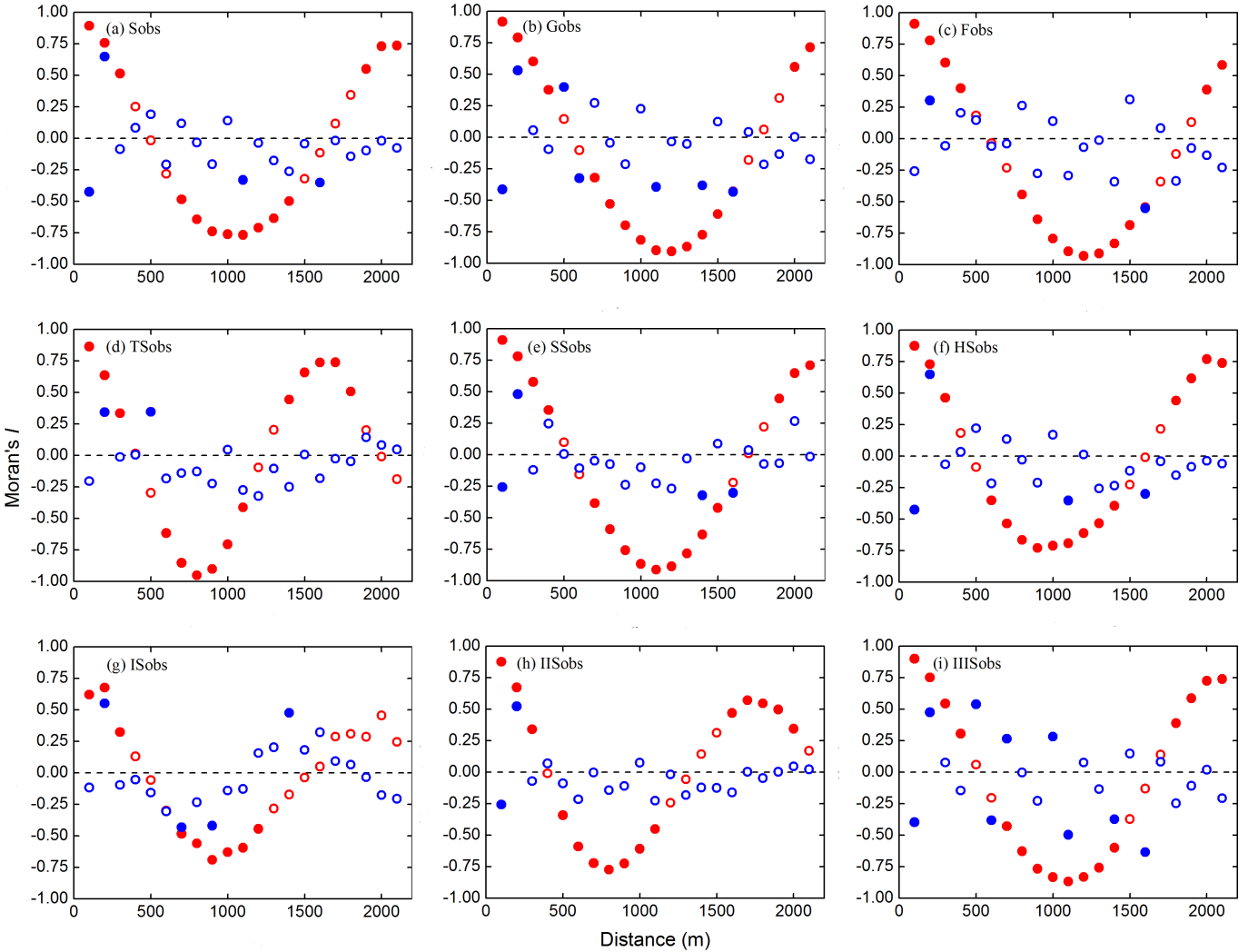
**

**Figure S7** Correlograms of ordinary least squares (red dots) and simultaneous autoregressive (blue dots) model residuals for the relationship between observed taxon richness and elevation. Filled dots indicate the significant spatial autocorrelation (*P*<.05). For abbreviations, see Figure S1.


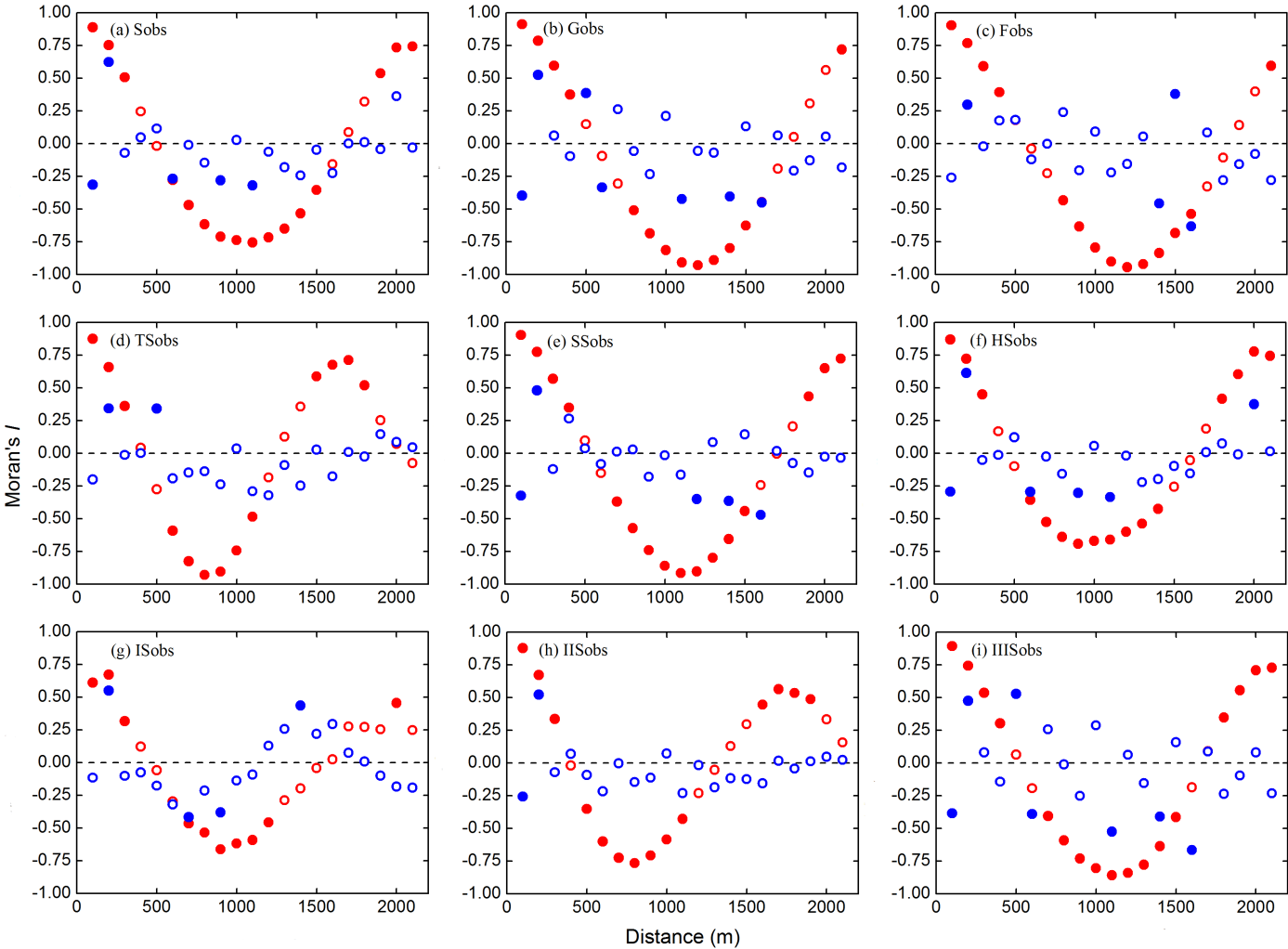


**Figure S8** Correlograms of ordinary least squares (red dots) and simultaneous autoregressive (blue dots) model residuals for the relationship between observed taxon richness and mean annual temperature. Filled dots indicate the significant spatial autocorrelation (*P*<.05). For abbreviations, see Figure S1.


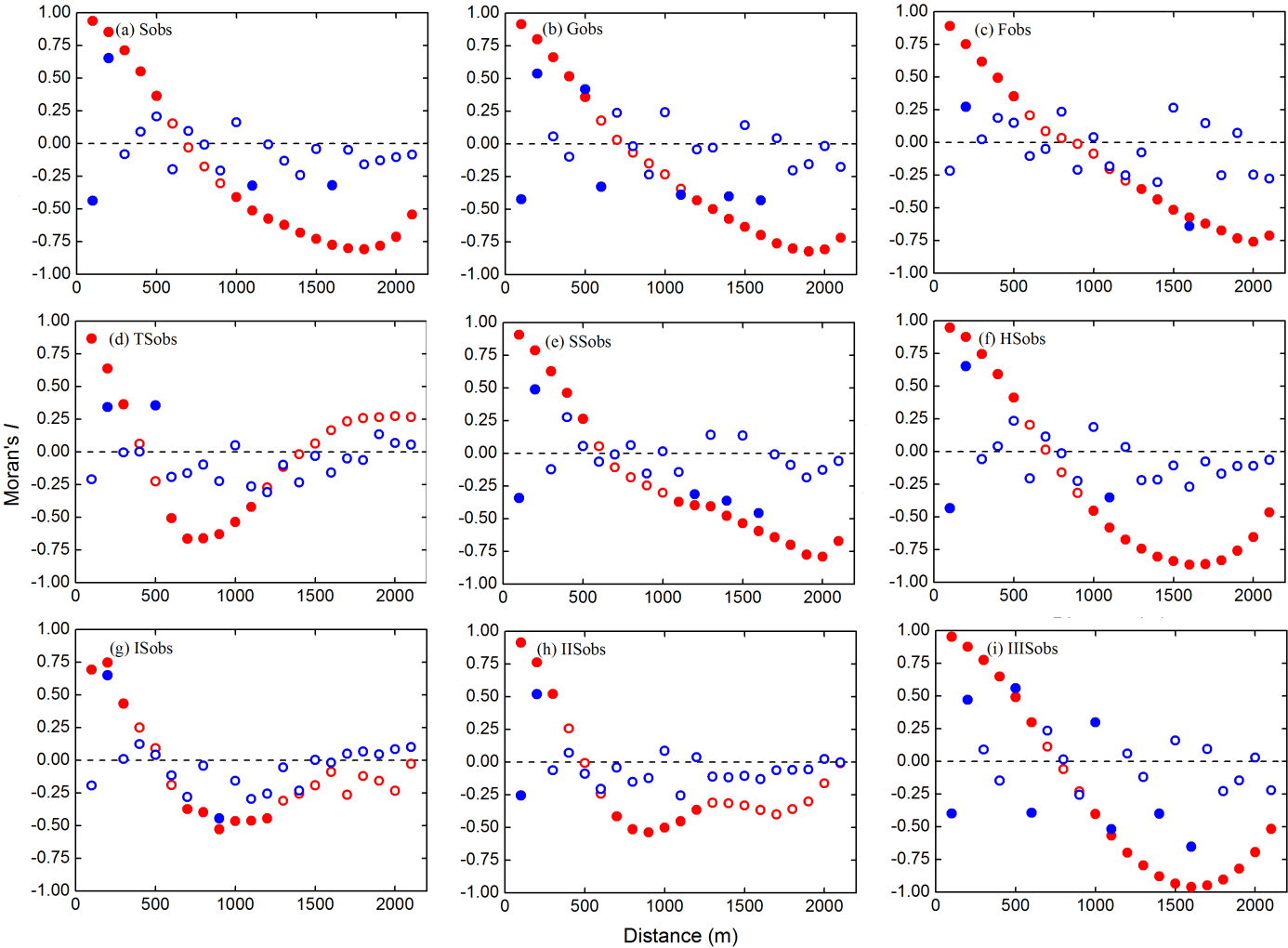


**Figure S9** Correlograms of ordinary least squares (red dots) and simultaneous autoregressive (blue dots) model residuals for the relationship between observed taxon richness and mean annual precipitation. Filled dots indicate the significant spatial autocorrelation (*P*<.05). For abbreviations, see Figure S1.


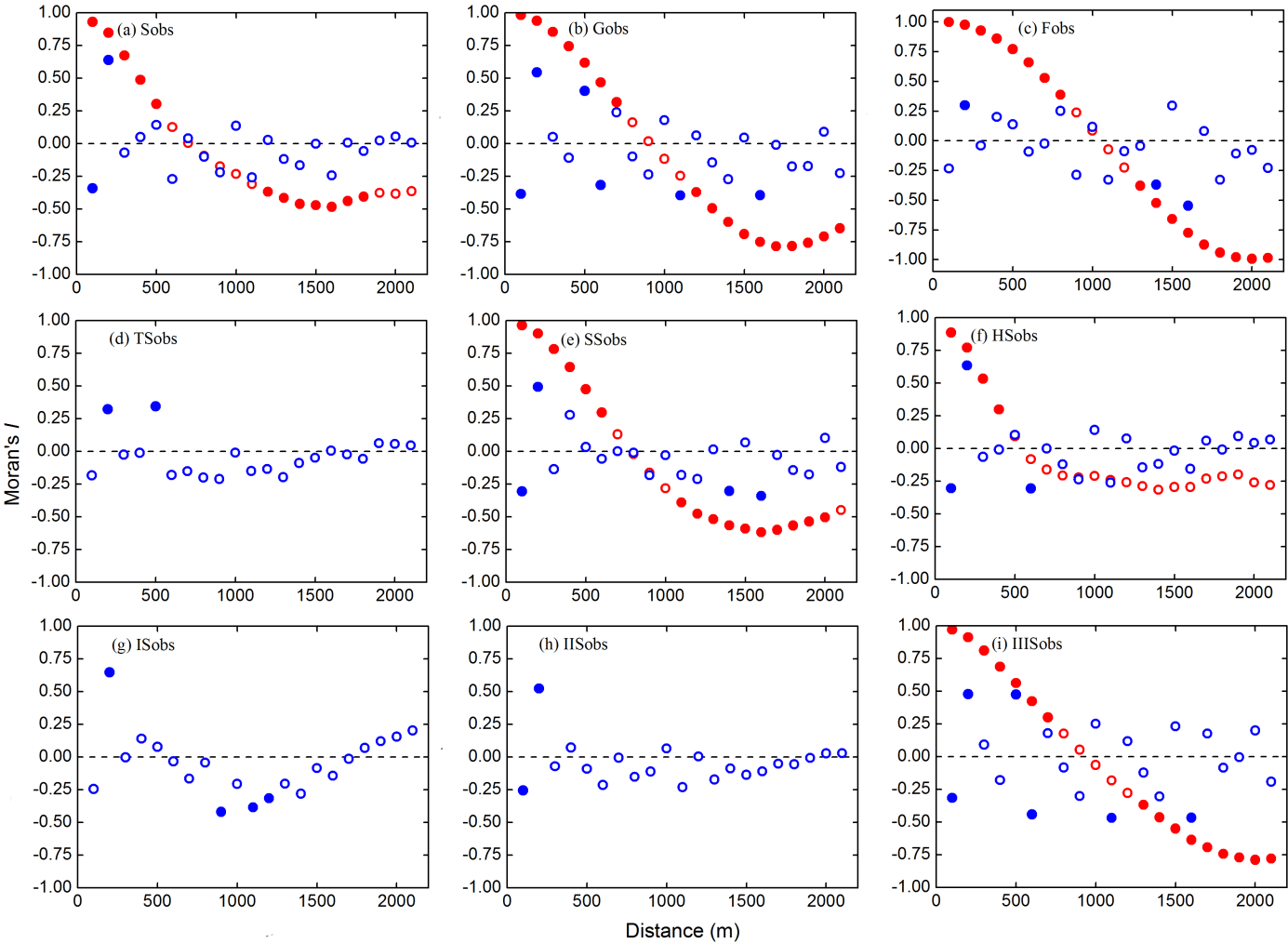


**Figure S10** Correlograms of ordinary least squares (red dots) and simultaneous autoregressive (blue dots) model residuals for the relationship between observed taxon richness and prediction from mid-domain effect. Non-significant regressions are not shown in the figures. Filled dots indicate the significant spatial autocorrelation (*P*<.05). For abbreviations, see Figure S1.


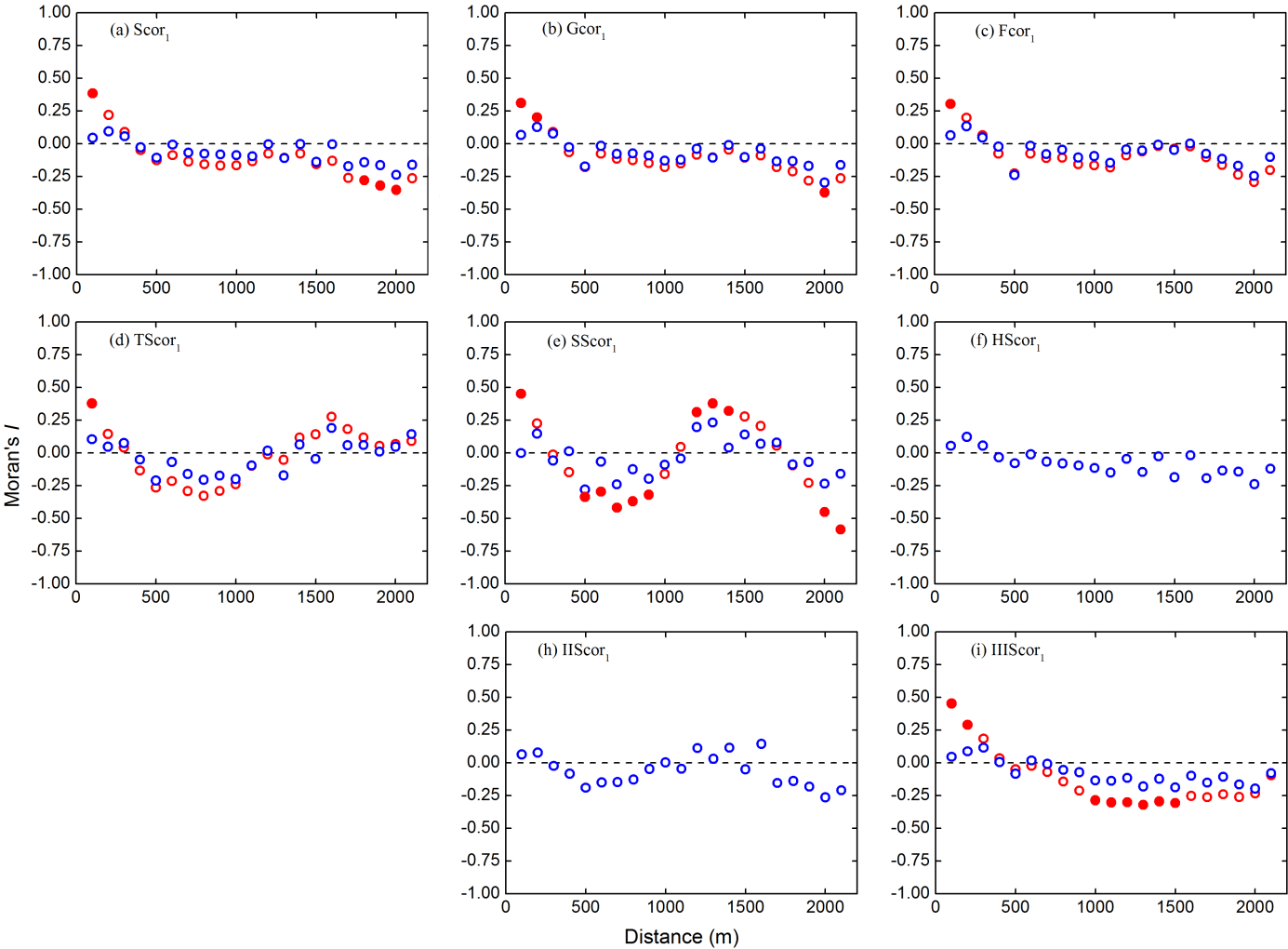


**Figure S11** Correlograms of ordinary least squares (red dots) and simultaneous autoregressive (blue dots) model residuals for the relationship between area-corrected taxon richness achieved by method 1 and elevation. Non-significant regressions are not shown in the figures. Filled dots indicate the significant spatial autocorrelation (*P*<.05). For abbreviations, see Figure S5.


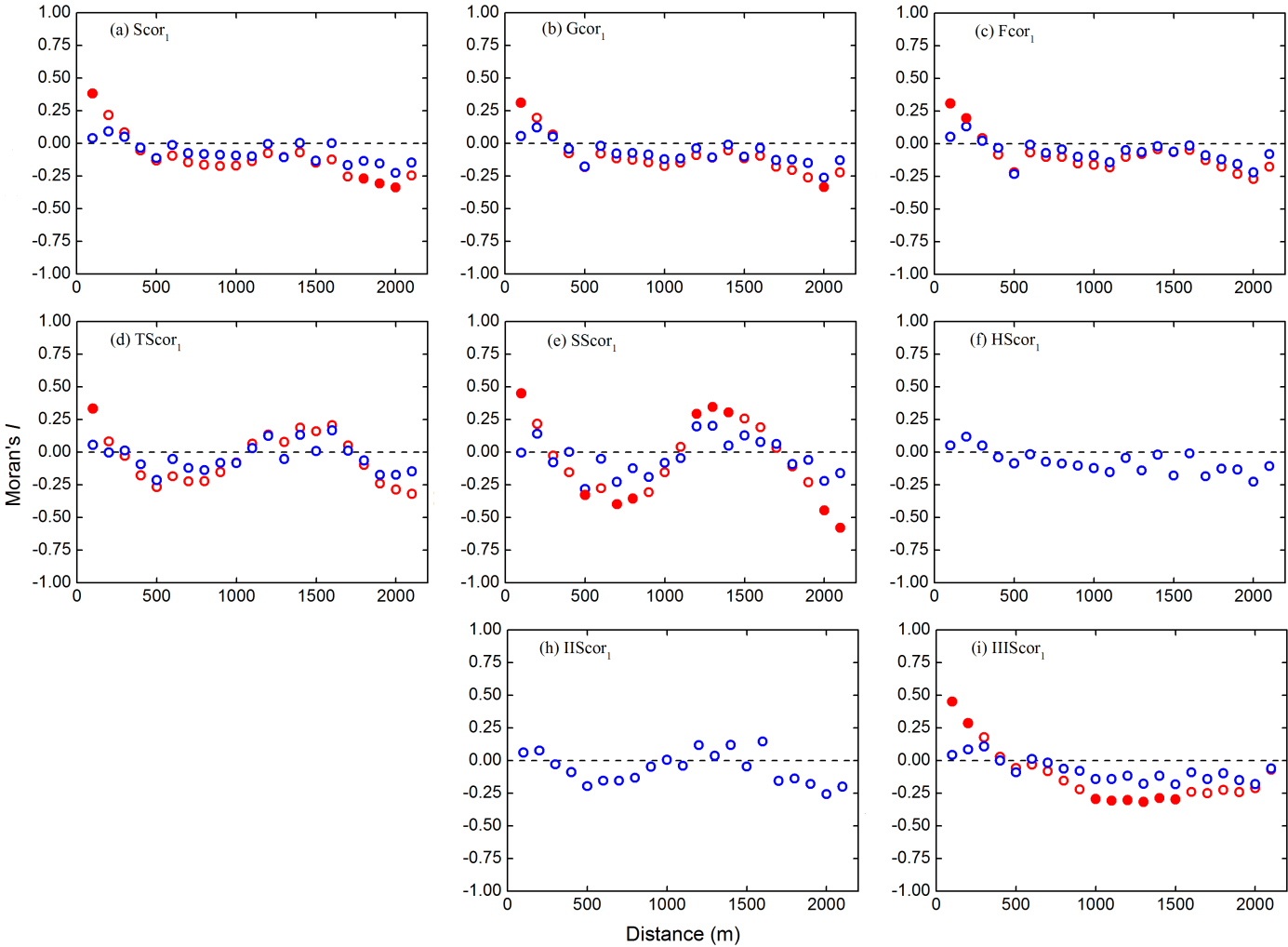


**Figure S12** Correlograms of ordinary least squares (red dots) and simultaneous autoregressive (blue dots) model residuals for the relationship between area-corrected taxon richness achieved by method 1 and mean annual temperature. Non-significant regressions are not shown in the figures. Filled dots indicate the significant spatial autocorrelation (*P*<.05). For abbreviations, see Figure S5.


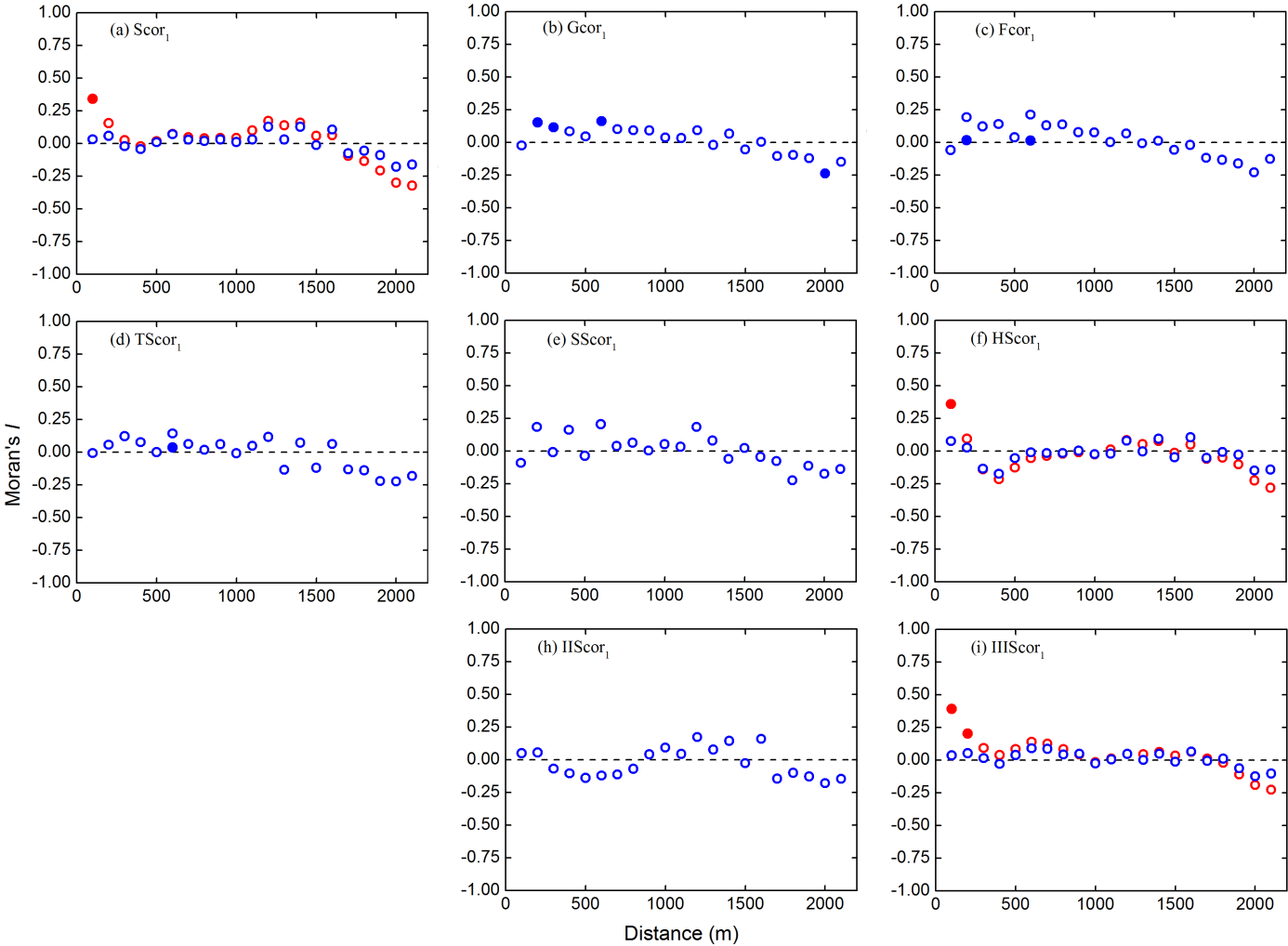


**Figure S13** Correlograms of ordinary least squares (red dots) and simultaneous autoregressive (blue dots) model residuals for the relationship between area-corrected taxon richness achieved by method 1 and mean annual precipitation. Non-significant regressions are not shown in the figures. Filled dots indicate the significant spatial autocorrelation (*P*<.05). For abbreviations, see Figure S5.


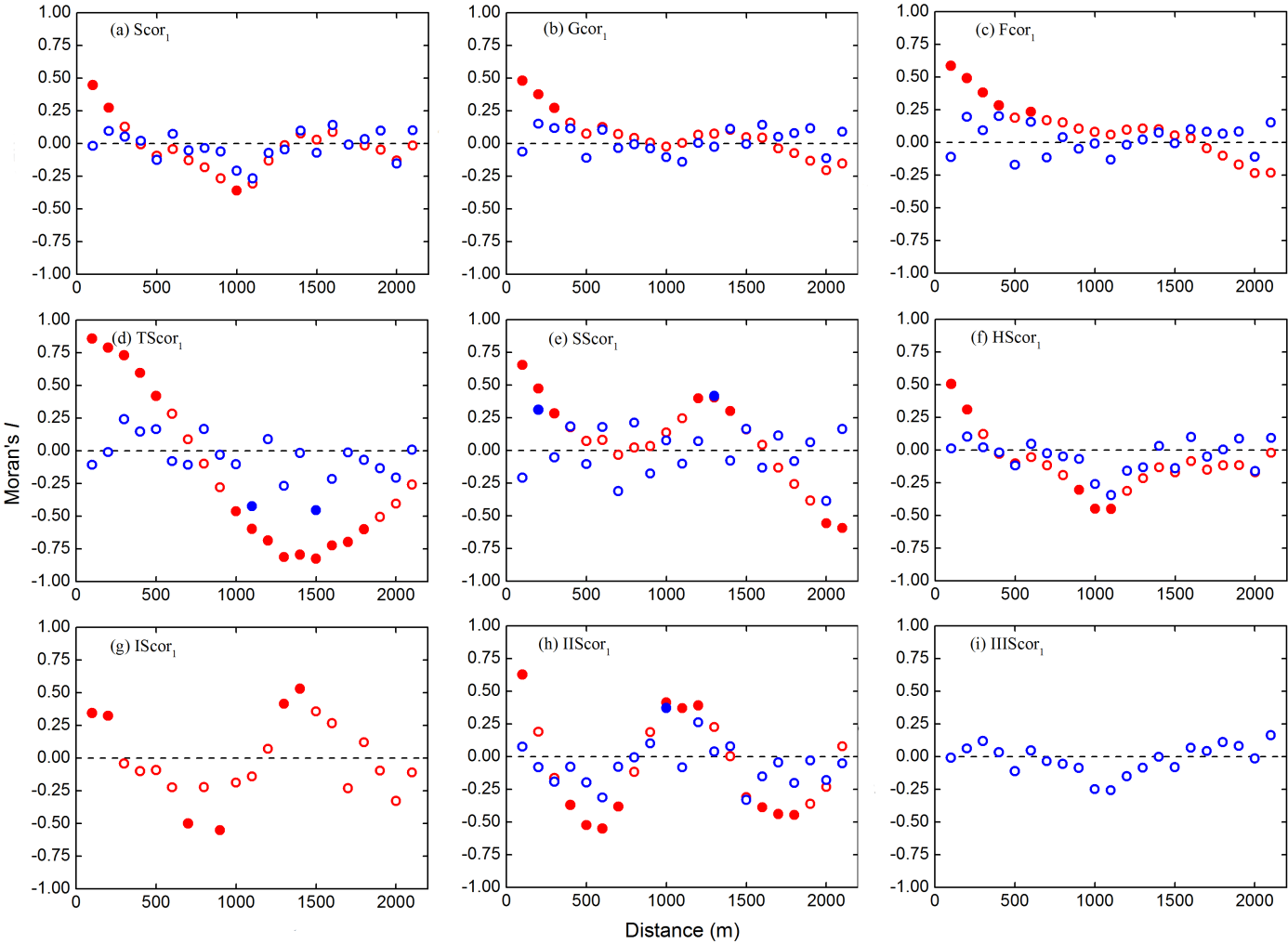


**Figure S14** Correlograms of ordinary least squares (red dots) and simultaneous autoregressive (blue dots) model residuals for the relationship between area-corrected taxon richness achieved by method 1 and prediction from mid-domain effect. Non-significant regressions are not shown in the figures. Filled dots indicate the significant spatial autocorrelation (*P*<.05). For abbreviations, see Figure S5.


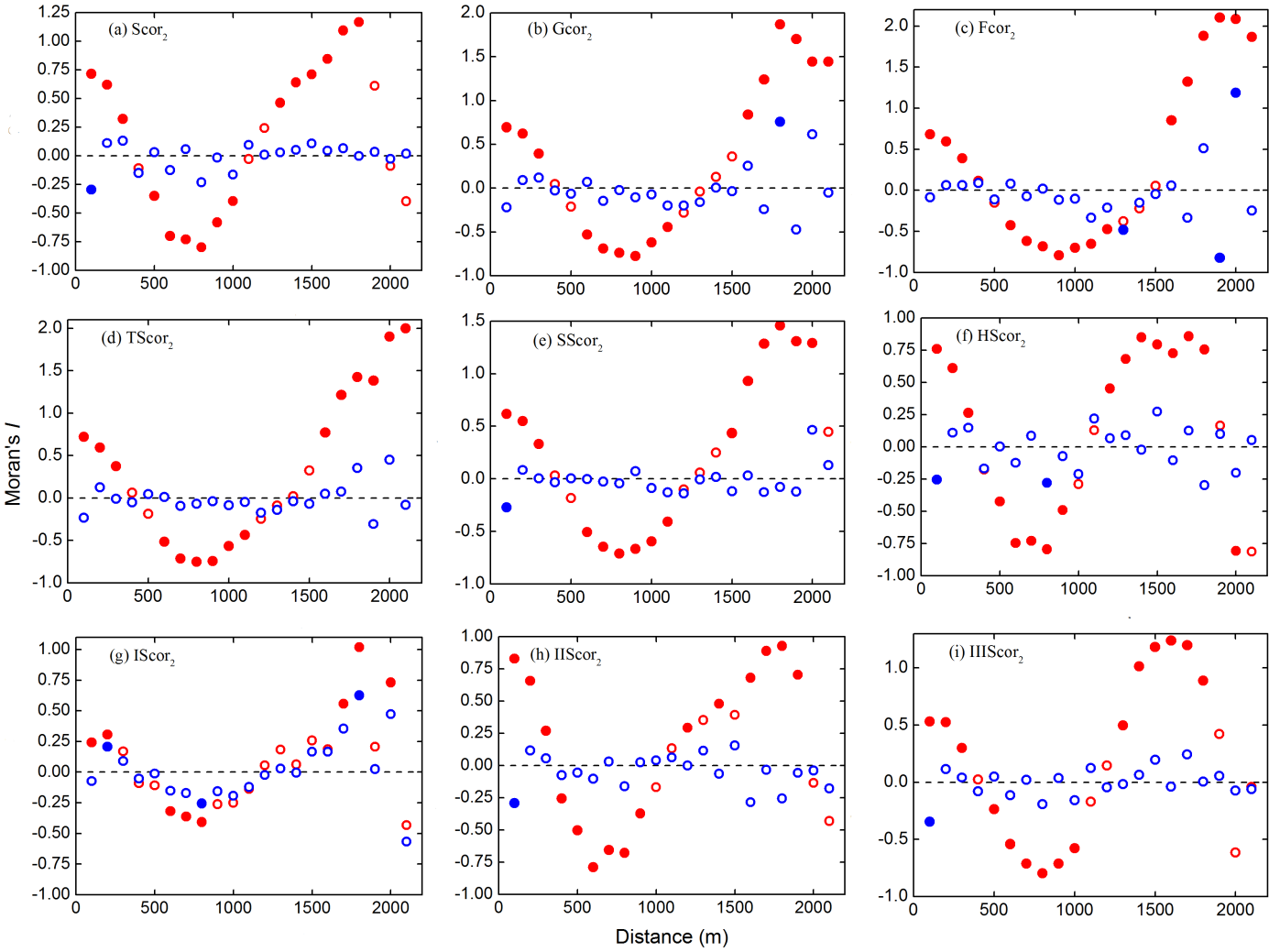


**Figure S15** Correlograms of ordinary least squares (red dots) and simultaneous autoregressive (blue dots) model residuals for the relationship between area-corrected taxon richness 2 and elevation. Filled dots indicate the significant spatial autocorrelation (*P*<.05). For abbreviations, see Figure S6.


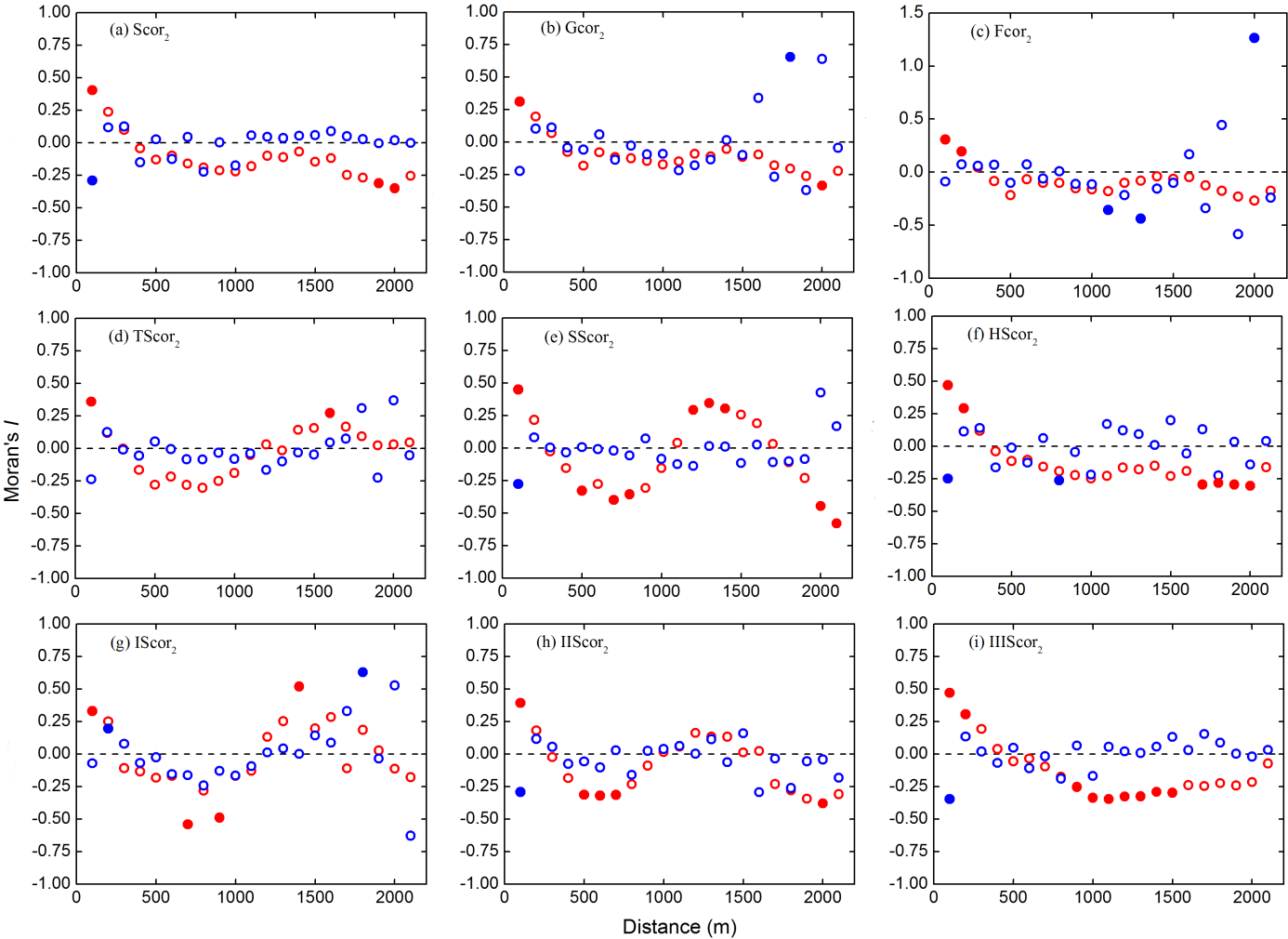


**Figure S16** Correlograms of ordinary least squares (red dots) and simultaneous autoregressive (blue dots) model residuals for the relationship between area-corrected taxon richness achieved by method 2 and mean annual temperature. Filled dots indicate the significant spatial autocorrelation (*P*<.05). For abbreviations, see Figure S6.


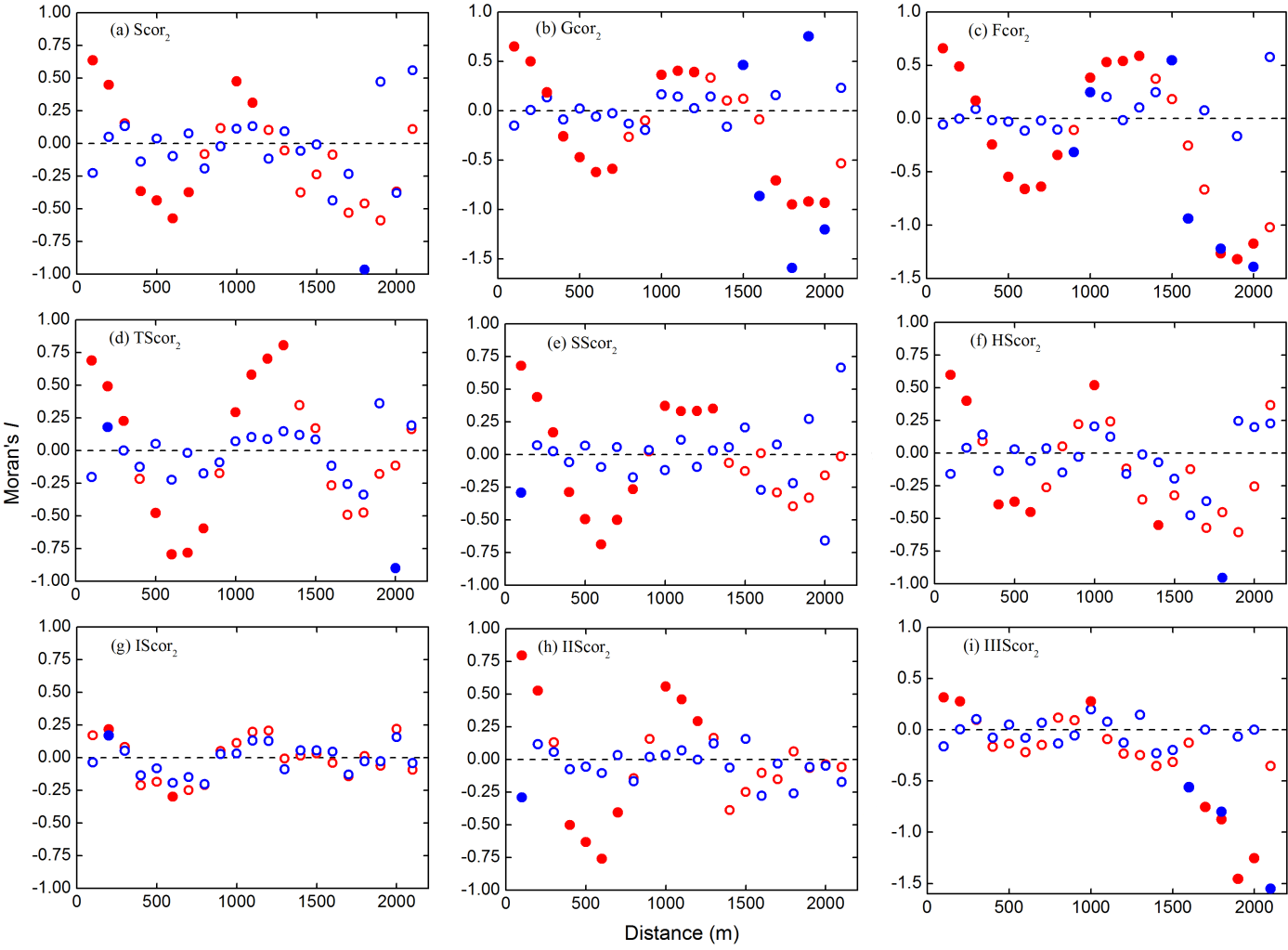


**Figure S17** Correlograms of ordinary least squares (red dots) and simultaneous autoregressive (blue dots) model residuals for the relationship between area-corrected taxon richness achieved by method 2 and mean annual precipitation. Filled dots indicate the significant spatial autocorrelation (*P*<.05). For abbreviations, see Figure S6.


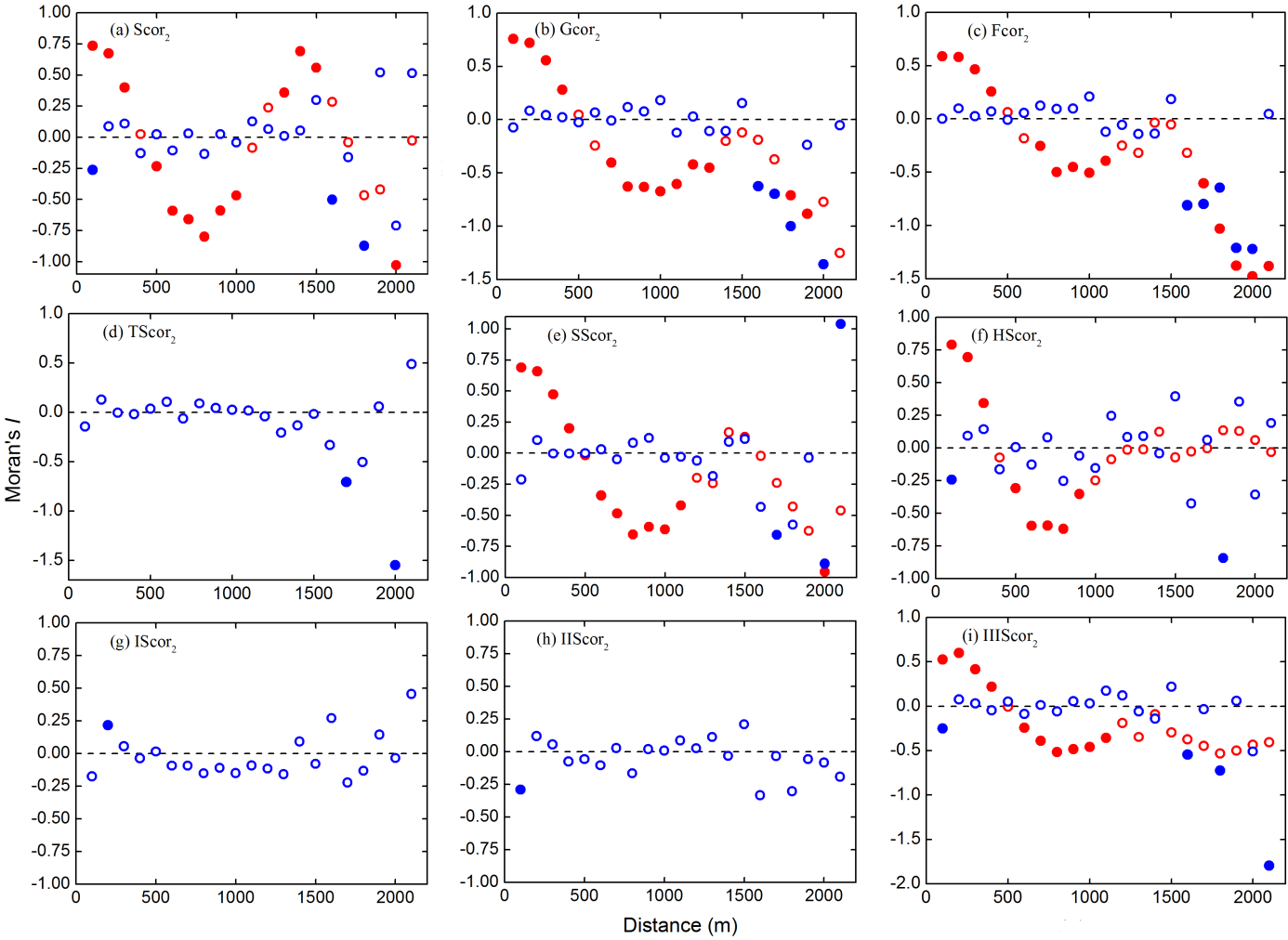


**Figure S18** Correlograms of ordinary least squares (red dots) and simultaneous autoregressive (blue dots) model residuals for the relationship between area-corrected taxon richness achieved by method 2 and prediction from mid-domain effect. Non-significant regressions are not shown in the figures. Filled dots indicate the significant spatial autocorrelation (*P*<.05). For abbreviations, see Figure S6.

**Table S1.** Species–area relationships using untransformed variables (species richness versus area), semi-log transformed variables (species richness versus log area) and log–log transformed variables (log species richness versus log area). All ordinary least squares regression models show that area effects are significant (*p*<.001). The best models are selected based on the minimum second-order Akaike’s information criterion (△AIC_C_>2), which are shown in the boldface type.

| **Observed taxon richness** | ***n*** | ***r^2^*** | | |
| --- | --- | --- | --- | --- |
|  |  | **Untransformed** | **Semi-log transformed** | **Semi-log transformed** |
| Seed plant species richness | 37 | 0.960 | 0.751 | **0.937** |
| Seed plant genus richness | 37 | 0.933 | 0.830 | **0.919** |
| Seed plant family richness | 37 | 0.822 | 0.845 | **0.875** |
| Tree species richness | 30 | 0.734 | 0.536 | **0.531** |
| Shrub species richness | 37 | 0.947 | 0.742 | **0.947** |
| Herb species richness | 37 | 0.916 | 0.722 | **0.926** |
| Group I species richness | 31 | 0.799 | 0.511 | **0.863** |
| Group II species richness | 34 | 0.817 | 0.514 | **0.892** |
| Group III species richness | 37 | 0.952 | 0.860 | **0.930** |

Group I, species with range size <150 m; Group II, species with range size between 150 m and 500 m; Group III, species with range size >500 m; *n*, number of samples; *r^2^*, adjusted coefficients of determination.

**Table S2.** Ordinary least squares models fit observed taxon richness against the first- and second-order polynomials of four variables (elevation, mean annual temperature, mean annual precipitation, and prediction from mid-domain effect). Significant models are marked with asterisks *** (*p*<.001), ** (*p*<.01) and * (*p*<.05). The models with the lowest second-order Akaike’s information criterion (△AIC_C_>2) are shown in the boldface type, and the models with △AIC_C_<2 are underlined.

| **Observed taxon richness** |  | **Elevation** | | | | | | | |
| --- | --- | --- | --- | --- | --- | --- | --- | --- | --- |
|  |  | **First-order** | | |  | **Second-order** | | | |
|  | *n* | *S* | *C* | *r^2^* |  | *Q* | *S* | *C* | *r^2^* |
| Seed plant species richness | 37 | -7.5×10^-2^ | 5.2×10^2^ | 0.068 |  | -1.9×10^-4^ | 1.1 | -1.2×10^3^ | **0.623***** |
| Seed plant genus richness | 37 | -5.4×10^-2^ | 3.2×10^2^ | 0.198** |  | -8.3×10^-5^ | 4.8×10^-1^ | -4.3×10^2^ | **0.681***** |
| Seed plant family richness | 37 | -1.9×10^-2^ | 1.1×10^2^ | 0.338*** |  | -2.2×10^-5^ | 1.2×10^-1^ | -8.3×10 | **0.756***** |
| Tree species richness | 30 | -1.3×10^-2^ | 8.3×10 | 0.058 |  | -4.3×10^-5^ | 2.3×10^-1^ | -2.4×10^2^ | **0.627***** |
| Shrub species richness | 37 | -1.7×10^-2^ | 1.0×10^2^ | 0.135* |  | -3.0×10^-5^ | 1.7×10^-1^ | -1.7×10^2^ | **0.576***** |
| Herb species richness | 37 | -4.0×10^-2^ | 3.3×10^2^ | 0.026 |  | -1.4×10^-4^ | 8.7×10^-1^ | -9.7×10^2^ | **0.642***** |
| Group I species richness | 31 | -3.3×10^-3^ | 3.9×10 | -0.024 |  | -2.1×10^-5^ | 1.2×10^-1^ | -1.3×10^2^ | **0.361***** |
| Group II species richness | 34 | -1.3×10^-2^ | 1.1×10^2^ | -0.011 |  | -6.9×10^-5^ | 4.1×10^-1^ | -4.7×10^2^ | **0.436***** |
| Group III species richness | 37 | -4.9×10^-2^ | 3.5×10^2^ | 0.090* |  | -1.2×10^-4^ | 7.2×10^-1^ | -7.5×10^2^ | **0.748***** |
|  |  | **Mean annual temperature** | | | | | | | |
| Seed plant species richness | 37 | 1.4×10 | 1.6×10^2^ | 0.080* |  | -6.6 | 1.3×10^2^ | -1.0×10^2^ | **0.640***** |
| Seed plant genus richness | 37 | 1.0×10 | 6.5×10 | 0.218** |  | -2.8 | 5.9×10 | -4.5×10 | **0.689***** |
| Seed plant family richness | 37 | 3.6 | 2.2×10 | 0.361*** |  | -7.4×10^-1^ | 1.6×10 | -6.8 | **0.758***** |
| Tree species richness | 30 | 2.7 | 1.6×10 | 0.077 |  | -1.4 | 3.2×10 | -1.1×10^2^ | **0.617***** |
| Shrub species richness | 37 | 3.3 | 2.1×10 | 0.152* |  | -1.0 | 2.1×10 | -1.8×10 | **0.583***** |
| Herb species richness | 37 | 7.8 | 1.3×10^2^ | 0.034 |  | -5.0 | 9.3×10 | -6.3×10 | **0.666***** |
| Group I species richness | 31 | 7.4×10^-1^ | 2.2×10 | -0.019 |  | -7.3×10^-1^ | 1.5×10 | -2.4×10 | **0.372***** |
| Group II species richness | 34 | 2.6 | 4.5×10 | -0.005 |  | -2.4 | 4.8×10 | -9.6×10 | **0.455***** |
| Group III species richness | 37 | 9.3 | 1.1×10^2^ | 0.103* |  | -4.2 | 8.1×10 | -5.0×10 | **0.768***** |
|  |  | **Mean annual precipitation** | | | | | | | |
| Seed plant species richness | 37 | 5.3 | -4.8×10^3^ | 0.203** |  | -9.2×10^-2^ | 1.8×10^2^ | -8.9×10^4^ | 0.204** |
| Seed plant genus richness | 37 | 3.2 | -2.9×10^3^ | **0.369***** |  | -2.5×10^-3^ | 8.0 | -5.2×10^3^ | 0.350*** |
| Seed plant family richness | 37 | 9.7×10^-1^ | -8.7×10^2^ | 0.414*** |  | 9.1×10^-3^ | -1.6×10 | 7.4×10^3^ | 0.411*** |
| Tree species richness | 30 | 1.2 | -1.1×10^3^ | 0.586*** |  | -2.1×10^-2^ | 4.2×10 | -2.0×10^4^ | 0.627*** |
| Shrub species richness | 37 | 1.2 | -1.1×10^3^ | **0.350***** |  | -6.4×10^-3^ | 1.4×10 | -7.0×10^3^ | 0.335*** |
| Herb species richness | 37 | 3.0 | -2.6×10^3^ | 0.116* |  | -8.9×10^-2^ | 1.7×10^2^ | -8.4×10^4^ | 0.137* |
| Group I species richness | 31 | 6.7×10^-1^ | -6.1×10^2^ | 0.247** |  | -2.2×10^-2^ | 4.2×10 | -2.0×10^4^ | 0.305** |
| Group II species richness | 34 | 1.8 | -1.7×10^3^ | 0.222** |  | -6.0×10^-2^ | 1.2×10^2^ | -5.7×10^4^ | 0.287** |
| Group III species richness | 37 | 2.9 | -2.6×10^3^ | 0.178** |  | -4.9×10^-2^ | 9.7×10 | -4.8×10^4^ | 0.175* |
|  |  | **Mid-domain effect** | | | | | | | |
| Seed plant species richness | 37 | 2.0 | -2.1×10^2^ | 0.464*** |  | 1.3×10^-2^ | -3.4 | 2.0×10^2^ | **0.605***** |
| Seed plant genus richness | 37 | 1.3 | -3.8×10 | 0.470*** |  | 6.5×10^-3^ | -2.8×10^-1^ | 3.4×10 | 0.491*** |
| Seed plant family richness | 37 | 1.0 | 8.6×10^-1^ | **0.399***** |  | 6.4×10^-3^ | 4.4×10^-1^ | 1.1×10 | 0.387*** |
| Tree species richness | 30 | 1.3 | -1.0 | **0.066** |  | 4.7×10^-2^ | -1.1 | 2.3×10 | 0.044 |
| Shrub species richness | 37 | 1.9 | -3.1×10 | 0.365*** |  | 6.0×10^-2^ | -2.4 | 2.6×10 | **0.432***** |
| Herb species richness | 37 | 2.0 | -1.6×10^2^ | 0.521*** |  | 1.9×10^-2^ | -3.7 | 1.5×10^2^ | **0.699***** |
| Group I species richness | 31 | 2.5 | -2.6×10 | **0.018** |  | -2.4 | 8.6×10 | -6.6×10^2^ | 0.004 |
| Group II species richness | 34 | 2.1 | -4.2×10 | 0.027 |  | 1.0×10^-1^ | -5.5 | 5.4×10 | 0.017 |
| Group III species richness | 37 | 1.5 | -7.3×10 | 0.608*** |  | 1.0×10^-2^ | -1.6 | 8.4×10 | **0.727***** |

The quadratic coefficient, slope and constant are labeled “Q”, “S” and “C” respectively. For abbreviations, see Table S1.

**Table S3.** Ordinary least squares models fit area-corrected taxon richness achieved by method 1 against the first- and second-order polynomials of four variables (elevation, mean annual temperature, mean annual precipitation, and prediction from mid-domain effect). Significant models are marked with asterisks *** (*p*<.001), ** (*p*<.01) and * (*p*<.05). The models with the lowest second-order Akaike’s information criterion (△AIC_C_>2) are shown in the boldface type, and the models with △AIC_C_<2 are underlined. For abbreviations, see Table S1 and S2.

| **Area-corrected taxon richness 1** |  | **Elevation** | | | | | | | | |
| --- | --- | --- | --- | --- | --- | --- | --- | --- | --- | --- |
|  |  | **First-order** | | |  | **Second-order** | | | |  |
|  | *n* | *S* | *C* | *r^2^* |  | *Q* | *S* | *C* | *r^2^* |  |
| Seed plant species richness | 37 | -5.1×10^-2^ | 4.4×10^2^ | 0.114* |  | 2.9×10^-5^ | -2.4×10^-1^ | 7.1×10^2^ | 0.131* |  |
| Seed plant genus richness | 37 | -8.8×10^-2^ | 5.8×10^2^ | 0.384*** |  | 5.8×10^-5^ | -4.6×10^-1^ | 1.1×10^3^ | **0.533***** |  |
| Seed plant family richness | 37 | -8.7×10^-2^ | 5.8×10^2^ | 0.501*** |  | 5.5×10^-5^ | -4.4×10^-1^ | 1.1×10^3^ | **0.682***** |  |
| Tree species richness | 30 | -5.6×10^-2^ | 2.3×10^2^ | 0.606*** |  | 2.3×10^-5^ | -1.9×10^-1^ | 4.0×10^2^ | **0.657***** |  |
| Shrub species richness | 37 | -1.4×10^-2^ | 1.1×10^2^ | 0.442*** |  | 8.5×10^-6^ | -6.9×10^-2^ | 1.9×10^2^ | **0.585***** |  |
| Herb species richness | 37 | -1.2×10^-2^ | 2.4×10^2^ | **-0.013** |  | -1.1×10^-6^ | -4.6×10^-3^ | 2.3×10^2^ | -0.042 |  |
| Group I species richness | 31 | -1.6×10^-3^ | 2.8×10 | **-0.013** |  | 9.3×10^-7^ | -7.2×10^-3^ | 3.6×10 | -0.042 |  |
| Group II species richness | 34 | -2.3×10^-3^ | 1.9×10 | 0.043 |  | 1.5×10^-6^ | -1.2×10^-2^ | 3.1×10 | 0.037 |  |
| Group III species richness | 37 | -5.4×10^-2^ | 5.0×10^2^ | 0.124* |  | 2.7×10^-5^ | -2.3×10^-1^ | 7.5×10^2^ | 0.133* |  |
|  |  | **Mean annual temperature** | | | | | | | | |
| Seed plant species richness | 37 | 8.6 | 2.1×10^2^ | 0.097* |  | 7.9×10^-1^ | -4.9 | 2.4×10^2^ | 0.097 |  |
| Seed plant genus richness | 37 | 1.5×10 | 1.6×10^2^ | 0.358*** |  | 1.9 | -1.7×10 | 2.3×10^2^ | **0.483***** |  |
| Seed plant family richness | 37 | 1.5×10 | 1.7×10^2^ | 0.474*** |  | 1.8 | -1.6×10 | 2.4×10^2^ | **0.640***** |  |
| Tree species richness | 30 | 1.0×10 | -3.3×10 | 0.576*** |  | 7.2×10^-1^ | -5.0 | 2.8×10 | 0.618*** |  |
| Shrub species richness | 37 | 2.5 | 4.5×10 | 0.418*** |  | 2.8×10^-1^ | -2.4 | 5.6×10 | **0.548***** |  |
| Herb species richness | 37 | 1.8 | 1.9×10^2^ | **-0.018** |  | -2.0×10^-1^ | 5.2 | 1.8×10^2^ | -0.044 |  |
| Group I species richness | 31 | 2.7×10^-1^ | 2.1×10 | **-0.016** |  | 2.1×10^-2^ | -1.2×10^-1^ | 2.2×10 | -0.049 |  |
| Group II species richness | 34 | 3.9×10^-1^ | 7.8 | **0.032** |  | 3.7×10^-2^ | -2.9×10^-1^ | 9.9 | 0.013 |  |
| Group III species richness | 37 | 9.2 | 2.5×10^2^ | 0.105* |  | 7.2×10^-1^ | -3.1 | 2.8×10^2^ | 0.100 |  |
|  |  | **Mean annual precipitation** | | | | | | | | |
| Seed plant species richness | 37 | -2.4 | 2.5×10^3^ | 0.119* |  | 5.46×10^-2^ | -1.07×10^2^ | 5.25×10^4^ | 0.121* |  |
| Seed plant genus richness | 37 | -1.0 | 1.3×10^3^ | -0.002 |  | 9.37×10^-2^ | -1.81×10^2^ | 8.71×10^4^ | 0.048 |  |
| Seed plant family richness | 37 | -4.7×10^-1^ | 7.5×10^2^ | -0.021 |  | 1.17×10^-1^ | -2.24×10^2^ | 1.08×10^5^ | **0.110** |  |
| Tree species richness | 30 | 3.2×10^-1^ | -2.4×10^2^ | **-0.017** |  | 1.19×10^-2^ | -2.24×10 | 1.07×10^4^ | -0.048 |  |
| Shrub species richness | 37 | -6.2×10^-2^ | 1.3×10^2^ | -0.024 |  | 1.75×10^-2^ | -3.36×10 | 1.62×10^4^ | **0.069** |  |
| Herb species richness | 37 | -2.2 | 2.3×10^3^ | **0.221**** |  | 1.50×10^-2^ | -3.09×10 | 1.60×10^4^ | 0.202** |  |
| Group I species richness | 31 | 4.9×10^-2^ | -2.3×10 | **-0.021** |  | 7.09×10^-4^ | -1.31 | 6.27×10^2^ | -0.057 |  |
| Group II species richness | 34 | -7.6×10^-2^ | 8.4×10 | **0.017** |  | -1.84×10^-4^ | 2.77×10^-1^ | -8.48×10 | -0.015 |  |
| Group III species richness | 37 | -2.8 | 3.0×10^3^ | 0.172** |  | 6.81×10^-2^ | -1.33×10^2^ | 6.54×10^4^ | 0.189* |  |
|  |  | **Mid-domain effect** | | | | | | | | |
| Seed plant species richness | 37 | -3.9×10^-1^ | 3.8×10^2^ | 0.036 |  | 7.6×10^-3^ | -3.6 | 6.3×10^2^ | **0.188*** |  |
| Seed plant genus richness | 37 | -9.2×10^-1^ | 4.3×10^2^ | 0.132* |  | 1.1×10^-2^ | -3.6 | 5.5×10^2^ | 0.177* |  |
| Seed plant family richness | 37 | -2.5 | 4.3×10^2^ | 0.159** |  | 4.4×10^-2^ | -6.5 | 5.0×10^2^ | 0.153* |  |
| Tree species richness | 30 | -5.5 | 2.6×10^2^ | 0.629*** |  | 2.3×10^-1^ | -1.7×10 | 3.8×10^2^ | **0.738***** |  |
| Shrub species richness | 37 | -6.1×10^-1^ | 9.4×10 | 0.151* |  | 1.9×10^-2^ | -2.0 | 1.1×10^2^ | 0.162* |  |
| Herb species richness | 37 | -4.3×10^-2^ | 2.1×10^2^ | -0.028 |  | 1.1×10^-2^ | -3.2 | 3.9×10^2^ | **0.136*** |  |
| Group I species richness | 31 | -1.7 | 6.0×10 | **0.166*** |  | 5.7×10^-3^ | -1.9 | 6.2×10 | 0.137* |  |
| Group II species richness | 34 | -5.8×10^-1^ | 4.2×10 | 0.447*** |  | 2.7×10^-2^ | -2.5 | 6.7×10 | **0.582***** |  |
| Group III species richness | 37 | -3.4×10^-1^ | 3.9×10^2^ | 0.005 |  | 9.7×10^-3^ | -3.2 | 5.4×10^2^ | **0.095** |  |

**Table S4.** Ordinary least squares models fit area-corrected taxon richness achieved by method 2 against the first- and second-order polynomials of four variables (elevation, mean annual temperature, mean annual precipitation, and prediction from mid-domain effect). Significant models are marked with asterisks *** (*p*<.001), ** (*p*<.01) and * (*p*<.05). The models with the lowest second-order Akaike’s information criterion (△AIC_C_>2) are shown in the boldface type, and the models with △AIC_C_<2 are underlined. For abbreviations, see Table S1 and S2.

| **Area-corrected taxon richness 2** |  | **Elevation** | | | | | | | |
| --- | --- | --- | --- | --- | --- | --- | --- | --- | --- |
|  |  | **First-order** | | |  | **Second-order** | | | |
|  | *n* | *S* | *C* | *r^2^* |  | *Q* | *S* | *C* | *r^2^* |
| Seed plant species richness | 37 | -4.8×10^-3^ | 5.4×10^2^ | -0.028 |  | -3.2×10^-4^ | 1.9 | -2.1×10^3^ | **0.572***** |
| Seed plant genus richness | 37 | -5.2×10^-2^ | 4.2×10^2^ | 0.085** |  | -1.2×10^-4^ | 6.9×10^-1^ | -6.4×10^2^ | **0.621***** |
| Seed plant family richness | 37 | -2.1×10^-2^ | 1.4×10^2^ | 0.266*** |  | -2.7×10^-5^ | 1.4×10^-1^ | -8.6×10 | **0.671***** |
| Tree species richness | 37 | -3.1×10^-2^ | 1.6×10^2^ | 0.204* |  | -3.6×10^-5^ | 1.8×10^-1^ | -1.5×10^2^ | **0.455***** |
| Shrub species richness | 37 | -1.8×10^-2^ | 1.4×10^2^ | 0.054 |  | -4.7×10^-5^ | 2.6×10^-1^ | -2.6×10^2^ | **0.510***** |
| Herb species richness | 37 | 4.4×10^-2^ | 2.4×10^2^ | -0.002 |  | -2.3×10^-4^ | 1.4 | -1.7×10^3^ | **0.610***** |
| Group I species richness | 37 | -9.3×10^-3^ | 9.6×10 | -0.010 |  | -3.7×10^-5^ | 2.1×10^-1^ | -2.2×10^2^ | **0.219**** |
| Group II species richness | 37 | -8.5×10^-3^ | 1.5×10^2^ | -0.025 |  | -1.1×10^-4^ | 6.2×10^-1^ | -7.4×10^2^ | **0.349***** |
| Group III species richness | 37 | 1.3×10^-2^ | 2.9×10^2^ | -0.023 |  | -1.7×10^-4^ | 1.0 | -1.2×10^3^ | **0.763***** |
|  |  | **Mean annual temperature** | | | | | | | |
| Seed plant species richness | 37 | 1.7 | 5.1×10^2^ | -0.028 |  | -1.3×10 | 2.5×10^2^ | -6.4×10^2^ | **0.643***** |
| Seed plant genus richness | 37 | 1.0×10 | 1.6×10^2^ | 0.098* |  | -4.9 | 1.1×10^2^ | -2.9×10^2^ | **0.687***** |
| Seed plant family richness | 37 | 4.0 | 4.2×10 | 0.288*** |  | -1.1 | 2.5×10 | -5.5×10 | **0.728***** |
| Tree species richness | 37 | 6.0 | 4.9 | 0.226** |  | -1.5 | 3.5×10 | -1.3×10^2^ | **0.517***** |
| Shrub species richness | 37 | 3.6 | 5.3×10 | 0.065 |  | -1.9 | 4.2×10 | -1.2×10^2^ | **0.584***** |
| Herb species richness | 37 | -7.8 | 4.5×10^2^ | -0.003 |  | -9.2 | 1.8×10^2^ | -3.9×10^2^ | **0.674***** |
| Group I species richness | 37 | 1.9 | 4.9×10 | -0.005 |  | -1.5 | 3.2×10 | -8.8×10 | **0.261**** |
| Group II species richness | 37 | 2.0 | 1.1×10^2^ | -0.024 |  | -4.2 | 8.7×10 | -2.8×10^2^ | **0.411***** |
| Group III species richness | 37 | -2.2 | 3.5×10^2^ | -0.024 |  | -6.8 | 1.3×10^2^ | -2.7×10^2^ | **0.830***** |
|  |  | **Mean annual precipitation** | | | | | | | |
| Seed plant species richness | 37 | 3.9 | -3.3×10^3^ | 0.099* |  | -5.4×10^-1^ | 1.0×10^3^ | -5.0×10^5^ | **0.619***** |
| Seed plant genus richness | 37 | 3.1 | -2.7×10^3^ | 0.427*** |  | -1.7×10^-1^ | 3.3×10^2^ | -1.6×10^5^ | **0.724***** |
| Seed plant family richness | 37 | 9.6×10^-1^ | -8.4×10^2^ | 0.662*** |  | -3.1×10^-2^ | 6.1×10 | -3.0×10^4^ | **0.821***** |
| Tree species richness | 37 | 1.5 | -1.4×10^3^ | 0.570*** |  | -4.7×10^-2^ | 9.3×10 | -4.5×10^4^ | **0.697***** |
| Shrub species richness | 37 | 1.2 | -1.0×10^3^ | 0.360*** |  | -7.6×10^-2^ | 1.5×10^2^ | -7.1×10^4^ | **0.712***** |
| Herb species richness | 37 | 1.3 | -8.6×10^2^ | -0.003 |  | -4.2×10^-1^ | 8.0×10^2^ | -3.9×10^5^ | **0.578***** |
| Group I species richness | 37 | 8.6×10^-1^ | -7.6×10^2^ | 0.150* |  | -5.8×10^-2^ | 1.1×10^2^ | -5.4×10^4^ | **0.311***** |
| Group II species richness | 37 | 1.8 | -1.6×10^3^ | 0.125* |  | -1.9×10^-1^ | 3.6×10^2^ | -1.8×10^5^ | **0.480***** |
| Group III species richness | 37 | 1.3 | -9.1×10^2^ | 0.029 |  | -2.9×10^-1^ | 5.7×10^2^ | -2.7×10^5^ | **0.699***** |
|  |  | **Mid-domain effect** | | | | | | | |
| Seed plant species richness | 37 | 3.1 | -4.3×10^2^ | 0.421*** |  | 1.6×10^-2^ | -5.3 | 5.4×10^2^ | 0.463*** |
| Seed plant genus richness | 37 | 1.2 | 4.0×10 | 0.235** |  | -7.4×10^-3^ | 3.5 | -1.1×10^2^ | 0.235** |
| Seed plant family richness | 37 | 5.5×10^-1^ | 4.7×10 | 0.076 |  | -3.4×10^-2^ | 4.2 | -4.2×10 | 0.152* |
| Tree species richness | 37 | 2.1 | -1.2×10 | **0.048** |  | -4.3×10^-2^ | 4.8 | -5.0×10 | 0.022 |
| Shrub species richness | 37 | 2.4 | -3.8×10 | **0.212**** |  | 4.2×10^-2^ | -1.3 | 3.7×10 | 0.197** |
| Herb species richness | 37 | 3.4 | -3.8×10^2^ | 0.531*** |  | 3.1×10^-2^ | -8.0 | 5.5×10^2^ | **0.634***** |
| Group I species richness | 37 | -1.1×10 | 3.2×10^2^ | **-0.026** |  | -7.6×10^-1^ | 2.4×10 | -8.2×10 | -0.056 |
| Group II species richness | 37 | 6.1 | -2.2×10^2^ | **0.014** |  | 2.7 | -2.5×10^2^ | 5.8×10^3^ | -0.008 |
| Group III species richness | 37 | 1.9 | -1.1×10^2^ | 0.638*** |  | 1.0×10^-2^ | -1.9 | 1.9×10^2^ | **0.698***** |
